# Anaerobic Methanotrophic Archaea of the ANME-2d Cluster Are Active in a Low-sulfate, Iron-rich Freshwater Sediment

**DOI:** 10.3389/fmicb.2017.00619

**Published:** 2017-04-12

**Authors:** Hannah S. Weber, Kirsten S. Habicht, Bo Thamdrup

**Affiliations:** Nordic Center for Earth Evolution and Department of Biology, University of Southern DenmarkOdense, Denmark

**Keywords:** anaerobic oxidation of methane, ANME-2d, RNA stable isotope probing, freshwater sediment, low-sulfate iron-rich natural environment

## Abstract

ANaerobic MEthanotrophic (ANME) archaea remove the greenhouse gas methane from anoxic environments and diminish its flux to the atmosphere. High methane removal efficiencies are well documented in marine environments, whereas anaerobic oxidation of methane (AOM) was only recently indicated as an important methane sink in freshwater systems. Freshwater AOM-mediating microorganisms lack taxonomic identification and only little is known about metabolic adaptions to prevailing biogeochemical conditions. One of the first study sites providing information about AOM activity in freshwater sediment is Lake Ørn, a low-sulfate, iron-rich Danish lake. With the aim to identify freshwater AOM-mediating archaea, we incubated AOM-active anoxic, nitrate-free freshwater sediment from Lake Ørn with ^13^C-labeled methane (^13^C_CH4_) and ^13^C-labeled bicarbonate (^13^C_DIC_) and followed the assimilation of ^13^C into RNA by stable isotope probing. While AOM was active, ^13^C_CH4_ and probably also ^13^C_DIC_ were incorporated into uncultured archaea of the *Methanosarcinales*-related cluster ANME-2d, whereas other known ANME lineages were not detected. This finding strongly suggests that ANME-2d archaea perform AOM coupled to sulfate and/or iron reduction and may have the capability of mixed assimilation of CH_4_ and DIC. ANME-2d archaea may thus play an important role in controlling methane emissions from nitrate-depleted and low-sulfate freshwater systems.

## Introduction

As a major sink of the greenhouse gas methane (CH_4_), anaerobic oxidation of methane (AOM) is a significant regulator of the global methane cycle (Knittel and Boetius, 2009). Analysis of 16S rRNA sequences links AOM to three distinct clusters of anaerobic methanotrophic archaea (ANaerobic MEthane oxidizers) named ANME-1, -2, and -3 that fall into the methanogenic orders *Methanomicrobiales* and *Methanosarcinales* (Knittel and Boetius, 2009) and in the class *Methanomicrobia*. The different ANME groups are related to each other with phylogenetic similarities as low as 75–92% (based on 16S rRNA sequences; Knittel and Boetius, 2009) and inhabit a wide range of anoxic environments that differ markedly in their biogeochemical conditions. ANME strains were reported to be abundant in anoxic marine environments such as sulfate-methane-transition-zones (SMTZs), cold and hot seeps, the deep biosphere, and anoxic marine water columns, and were also, albeit rarely, detected in soils, aquifers and freshwater sediments (Knittel and Boetius, 2009 and references therein).

All ANME strains oxidize methane using the reverse methanogenesis pathway (Hallam et al., 2004). Reducing equivalents resulting from anaerobic methane oxidation are transferred to either sulfate (Hinrichs et al., 1999; Boetius et al., 2000; Orphan et al., 2002), nitrate (Haroon et al., 2013), or iron and possibly manganese (Beal et al., 2009, 2011; Ettwig et al., 2016; Scheller et al., 2016). The transfer mechanisms of the reducing equivalents in ANME strains are not fully understood and different concepts have evolved according to the different electron acceptors. During sulfate-dependent AOM, ANME strains were often observed in consortia with sulfate-reducing bacteria (Boetius et al., 2000) and currently three concepts allowing reducing equivalent transfer are discussed: (1) an obligate syntrophic process between the partners involving an interspecies electron carrier (e.g., Meulepas et al., 2010), (2) direct electron transfer by conductive pili (nanowires) providing cell-to-cell contact (McGlynn et al., 2015; Wegener et al., 2015) or (3) ANME carrying out both methane oxidation and sulfate reduction and excreting zero-valent sulfur compounds that are further disproportionated by the associated bacterial partners (Milucka et al., 2012). In contrast to this puzzling diversity of pathways, a nitrate-reducing ANME strain named ‘*Candidatus* Methanoperedens nitroreducens’ was shown to have the enzymatic capability to perform reverse methanogenesis while reducing nitrate on its own (Haroon et al., 2013). This strain is affiliated with the subgroup ANME-2d, also known as AAA (AOM-Associated Archaea), which was previously found in diverse environments with AOM activity but not conclusively linked to AOM (Knittel and Boetius, 2009). A coupling of AOM to the reduction of soluble ferric complexes was recently observed in an ANME-2 enrichment (Scheller et al., 2016) and a coupling to solid iron or manganese oxide reduction was suggested several times based on biogeochemical evidence (Beal et al., 2009; Sivan et al., 2011; Riedinger et al., 2014; Egger et al., 2015; Oni et al., 2015). Most recently, a strain closely related to ‘*Cand.* M. nitroreducens’ from a freshwater enrichment culture was shown to couple AOM to the reduction of soluble and nanoparticulate forms of ferric iron (Ettwig et al., 2016).

Our knowledge about AOM and the microorganisms involved derives almost exclusively from investigations of marine environments. In anoxic freshwater environments, sulfate-dependent AOM has often been assumed to be insignificant due to low sulfate concentrations (<1 mmol L^-1^), but recent studies have shown that AOM can provide an efficient barrier to methane emission from lake sediment even at sulfate concentrations < 0.2 mmol L^-1^ (Norði et al., 2013; Weber et al., 2016). In addition to the potential direct coupling of AOM to iron oxide reduction, AOM in low-sulfate environments could at least partially be coupled to sulfate reduction (Norði et al., 2013; Weber et al., 2016) with sulfate being recycled in the anoxic sediment by an iron-driven cryptic sulfur cycle (Riedinger et al., 2010; Holmkvist et al., 2011). Nitrate/nitrite-dependent AOM has also been documented in freshwater systems (Raghoebarsing et al., 2006; Deutzmann et al., 2014). There, nitrite-dependent AOM appears to be linked to the presence of bacteria of the NC10 clade (Ettwig et al., 2010; Deutzmann et al., 2014), while nitrate-dependent anaerobic methane oxidation might be carried out by the above-mentioned ‘*Cand.* Methanoperedens nitroreducens,’ although the distribution and role of this type of organism in natural systems remains to be investigated. Besides the ability to couple AOM to iron reduction shown in an enrichment culture (Ettwig et al., 2016), members of the ANME-2d or AAA lineage containing ‘*Cand.* M. nitroreducens’ were suggested to convey sulfate-dependent AOM in the sediment of the alpine Lake Cadagno (Schubert et al., 2011) and a natural freshwater gas source (Timmers et al., 2016). The identity of microorganisms coupling freshwater AOM to sulfur cycling at low-sulfate conditions and metal oxide reduction, remains puzzling, but is of high importance for elucidating the pathways of methane oxidation in freshwater systems and understanding how the metabolic constraints and adaptions of microbes involved in AOM affect the global significance of the process.

In the present study, we aimed at revealing the taxonomic identity of anaerobic methane oxidizers in the low-sulfate, iron-rich sediment of Danish Lake Ørn, where AOM activity is well documented (Norði et al., 2013; Weber et al., 2016). We incubated sediment from the AOM-active zone of Lake Ørn with ^13^C-labeled methane (^13^C_CH4_) and ^13^C-labeled bicarbonate (^13^C-Dissolved Inorganic Carbon: ^13^C_DIC_) and applied RNA stable isotope probing to identify archaea, which assimilated the ^13^C-labeled carbon sources. Additionally, we followed the turnover of sulfate and methane and determined sulfate reduction and anaerobic methane oxidation rates during incubations.

## Materials and Methods

### Study Site and Sediment Sampling

Lake Ørn is a shallow mesotrophic Lake with iron-rich sediment located in Jutland, Denmark. It has an area of 0.43 km^2^, an average water depth of 4 m and a maximum water depth of 10.5 m (Skovgaard, 2004). The stream Funder Å supplies the lake with a high input of iron in form of ochre (∼45 g Fe m^-2^ year^-1^ from 1989 to 2002; Skovgaard, 2004). Stratification is usually restricted to water depths greater than ∼5 m due to a high water flow and wind exposure.

Sediment sampling took place in April 2012 at the 4.5 m deep site near the center of the lake, for which the biogeochemistry was described previously (Norði et al., 2013; Weber et al., 2016). The water temperature was 8°C and the water column was oxygenated. Using a hand-operated Kajak sampler, three sediment cores (length = 30 cm, inner diameter = 7.5 cm) were retrieved for depth profiles of sulfate, methane, density/porosity and for RNA extraction. We dissected the cores obtained for depth profile characterization in 2 cm depth intervals under constant nitrogen flow within 2 days after sampling and subsampled for the different parameters as described below. To minimize a possible change of the biogeochemical depth profiles during storage, we air-bubbled the individual cores ∼2 cm above the sediment surface while keeping all cores in a tank filled with surface water from Lake Ørn.

Additionally, 30 cores were taken for RNA stable isotope probing incubation experiments and further treated as described in the following section.

### RNA Stable Isotope Probing (RNA-SIP) Incubation Experiments

RNA-SIP experiments were carried out in both core and slurry incubations. Both slurries and cores were subjected to five different treatments: addition of (1) ^13^C-bicarbonate (^13^C_DIC_), (2) ^12^C-bicarbonate (^12^C_DIC_), (3) ^13^C-methane (^13^C_CH4_), (4) ^12^C-methane (^12^C_CH4_), and (5) no addition as control. For core incubations, 24 cores were transferred immediately after sampling to a 5°C tempered water tank (total volume ∼0.2 m^3^), which was filled with surface water from Lake Ørn. All cores were kept at these conditions for the entire incubation time and were individually and continuously oxygenated by gentle air bubbling ∼1 cm above the sediment. The core liners contained holes (diameter = 0.2 cm), which were billowy winding around the liners (horizontal distance = 1 cm, vertical distance = 2 cm) and were double sealed with aquarium silicon (Dana Lim 579, ISO 11600) and poly-coated cloth tape (Tesa). ^13^C_CH4_ and ^12^C_CH4_ (both 99 atom-%), ^13^C_DIC_ and ^12^C_DIC_ (both 99 atom-%) were injected anoxically into the cores in 1 cm steps to 18 cm depth for methane and 25 cm depth for sodium bicarbonate, by means of a gas-tight syringe. Methane was added in increasing amounts with increasing sediment depth at days 1, 4, 7, and 15 corresponding to final added concentrations of ∼40 μmol L^-1^ (0–5 cm); ∼80 μmol L^-1^ (5–10 cm), ∼120 μmol L^-1^ (10–15 cm), and ∼260 μmol L^-1^ (15–20 cm), which corresponded to ∼50% of the initial methane concentrations and up to ∼80% of ^13^CH_4_ at the last time point. ^13^C_DIC_ and ^12^C_DIC_ was supplied from an anoxic stock solution (1 mol L^-1^) at days 1 and 14 to added concentrations of ∼1.4 mmol L^-1^ (0–5 cm), ∼1.7 mmol L^-1^ (5–15 cm), and ∼2 mmol L^-1^ (15–25 cm). δ^13^C_DIC_ measurements confirmed a range of ∼7 to ∼25 atom-% ^13^C_DIC_ in the DIC pool of the entire depth, whereas in the layer 10–15 cm, ^13^C_DIC_ was in a range of ∼13 to 21 atom-%.

The cores were incubated for 30 days in total and sampled after 3, 6, 14, and 30 days. For sampling, one control core, one ^12^C core, and one ^13^C core were sliced under constant nitrogen flow into 5 cm intervals to a sampling depth of 25 cm. At each time point, subsamples were taken to determine porosity, concentrations of sulfate and methane, to quantify rates of microbial sulfate reduction (SR) and anaerobic oxidation of methane (AOM), and for RNA extraction for RNA-SIP analysis.

For slurry SIP incubations, sediment from the depth interval 10–15 cm was extracted from six sediment cores under constant nitrogen flow, transferred into a glass bottle (Schott) and sealed with a butyl rubber stopper. The bottle was transferred into a nitrogen-filled glove bag and sediment was mixed 1:1 with N_2_-purged medium (50 μmol L^-1^ Na_2_SO_4_ and 3 mmol L^-1^ NaHCO_3_). Slurry was then distributed into 20 serum bottles (60 mL) that were closed with butyl rubber stoppers (kept under helium atmosphere prior to usage; De Brabandere et al., 2012) without headspace. By means of a gas-tight syringe, ^12^C_CH4_ was added at day 0 to all bottles through the butyl rubber stopper to a final concentration of 120 μmol L^-1^, simulating concentrations similar to the core incubations. One-third of the incubations received additional 60 μmol L^-1 12^C_CH4_ and one-third received additional 60 μmol L^-1 13^C_CH4_, which raised the total methane concentration to 180 μmol L^-1^ in these incubations. In the remaining 1/3 of the slurries, no further methane was added as they were used as controls. At days 3, 6, and 27 additional 60 μmol L^-1 12^C_CH4_ or ^13^C_CH4_ was added to the ^12^C_CH4_ and ^13^C_CH4_ incubations, respectively. For DIC slurry experiments, 1.3 mmol L^-1 12^C_DIC_ or ^13^C_DIC_ was added to ^12^C_DIC_ and ^13^C_DIC_ incubations, respectively, at days 1 and 27. δ^13^C_DIC_ measurements confirmed a range of ∼15 to ∼25 ^13^C_DIC_ atom-% in the DIC pool. All bottles were stored in darkness at 5°C and carefully inverted every few days. In total we had 20 slurries, which were successively sampled after 3, 6, 27, and 72 days. For sampling, one bottle from each treatment was opened in a nitrogen-flushed glove bag and sampled for measurements of the same biogeochemical, molecular and rate measurements as described for the cores.

### Gaseous, Solid, and Porewater Parameters

All cores and slurries were processed under constant nitrogen flow and at 4°C and subsamples were taken either by cut-off syringes or a spatula.

For porewater analysis, 15 mL centrifugation vials were filled completely, capped, centrifuged at 4°C at 4000 *g* for 10 min (Centrifuge 5810; Eppendorf) and the supernatant was filtered anoxically (glove bag filled with nitrogen) through a syringe filter (pore size = 0.22 μm; syringe, needle and filter flushed and stored in helium prior to usage). For analysis of sulfate, 1 ml porewater each was mixed with 20 μL 20% zinc acetate (ZnAc) and stored at -20°C. Sulfate concentrations were quantified via ion chromatography (Dionex ICS-1500, detector: DS6 Heated Conductivity Cell), using ultrapure water (“type 1”) containing 4.5 mmol L^-1^ Na_2_CO_3_ and 1.4 mmol L^-1^ NaHCO_3_, as eluent. The detection limit for sulfate was 1 μmol L^-1^. For DIC concentration and δ^13^C measurements, porewater was stored in 1.8 ml septum vials on 10 μL saturated mercury chloride (HgCl_2_) at 4°C. For analysis 300 μL sample was transferred into a 12 mL acid washed and helium-flushed Exetainer (Labco, High Wycombe, UK) and 50 μL of 85% phosphoric acid was added to drive CO_2_ into the headspace over an equilibration phase of 15 h. The ^13^C atom-% values were determined by IRMS, using the standards NBS 18-Calcite and IAEA-LSVEC Lithium Carbonate and an in-house bicarbonate standard (average standard deviation between samples = 0.1%).

To determine methane concentrations, 2 cm^3^ of sediment or slurry was subsampled directly into a 20 mL serum vial, prefilled with 5 mL 2.5% sodium hydroxide (NaOH) that was closed and shaken immediately and stored upside down for at least 24 h to drive the methane into the headspace. Methane concentrations were quantified by injecting 50–100 μL of the headspace into a gas chromatograph with an FID detector (Perkin Elmer). The porosity/density of sediment or slurry was determined by subsampling 1 cm^3^ sediment or slurry with a cut-off pipette tip into a pre-weighted centrifugation vial and the water content/porosity from the weight loss from after drying at 90°C for 48 h.

### Rates of Dissimilatory Sulfate Reduction (SR) and Anaerobic Oxidation of Methane (AOM)

For SR and AOM rates, 5 cm^3^ sediment or slurry was filled into glass tubes in triplicates or duplicates, respectively, closed with plunger and septum (stored under helium 6 weeks prior to sampling) and pre-incubated for 1 h before injection of radiotracers (Jørgensen, 1978).

For SR rates, 20 μL carrier-free ^35^SO_4_^2-^ tracer (100 kBq) sample^-1^ was injected through the septum, the tubes inverted and the samples incubated at 5°C in darkness for 24 h. The incubations were stopped by transferring the samples into 10 mL of 20% ZnAc. Controls (“killed controls”) were taken in parallel, in which sediment/slurry was transferred into 10 mL of 20% ZnAc after 24 h and thereafter tracer was added. All samples and controls were frozen immediately and stored at -20°C until analysis. After thawing, supernatants were separated from solid phases by centrifuging the samples at 500 g for 5 min and decanting. Supernatants were subsampled for determination of ^35^S in sulfate and reduced sulfur compounds were extracted via the cold chromium distillation for ^35^S determination (Kallmeyer et al., 2004; Røy et al., 2014).

For AOM rates, ^14^CH_4_ (dissolved in water; 20 μl; ∼0.5 kBq; supplied from American Radiolabeled Chemicals, cleaned with hopcalite and sodium hydroxide) was injected through the septum into the sediment or slurry, the tubes inverted and samples incubated at 5°C in darkness for 20 h. In order to stop incubations the samples were transferred into glass vials prefilled with 15 ml 2.5% (w/v) sodium hydroxide and immediately closed with butyl rubber stoppers, shaken and stored upside down. Methane concentrations were determined as described above and activities of ^14^CH_4_ and ^14^CO_2_ were quantified (Treude et al., 2005), using phenylethylamine and sodium hydroxide (1:1) as CO_2_ trap. Duplicates of “killed controls” were taken with each time point, in which tracer was injected after termination of microbial activity.

Radioactivity was measured on a QuantaSmart-4.00 Scintillation counter (count time: 10 min; coincidence time: 18 nsec; delay before burst: 75 nsec). The detection limits for the SR and AOM rate measurements were determined by subtracting the mean plus three times the standard deviation of the killed controls from the product pools, ^35^S sulfides and ^14^CO_2_, respectively.

### Subsampling, Extraction, and Quantification of RNA

For each time point and incubation treatment, ∼5 cm^3^ of sediment or slurry was frozen immediately after sampling in liquid nitrogen and stored at -80°C until further processing. RNA was extracted using the PowerSoil Total RNA Isolation Kit (MoBio) and further purified using the AllPrep DNA/RNA Mini Kit (Quiagen). RNA concentrations were quantified with the Quant-iT-RiboGreen RNA Assay Kit (Life Technologies).

### Caesium Trifluoroacetate (CsTFA) Density Gradient Ultracentrifugation and Fractionation

Caesium trifluoroacetate density gradient ultracentrifugation was applied as described previously (Whiteley et al., 2007). Briefly, extracted and purified RNA (∼600 ng in 24 μL nuclease-free ultrapure water) was mixed with a carefully adjusted density (1.8 ± 0.04 g mL^-1^) gradient medium containing CsTFA (4.64 mL sample^-1^), nuclease-free ultrapure water (0.936 mL sample^-1^) and formamide (0.198 mL sample^-1^). From this mixture, 5.1 mL was transferred into ultracentrifugation tubes (Quick-Seal, 5.2 mL, 13 × 51 mm; polyallomer, Beckmann Coulter) and closed by heat sealing. The tubes were centrifuged for 72 h (Beckmann Ultracentrifuge Optima XPN 80k RPM; Vertical rotor VTi 65,2) at 38400 rpm, 20°C with maximum acceleration and deceleration. After ultracentrifugation, the gradient was fractionated into 20 fractions by driving the gradient out at the bottom of the tube at a controlled constant flow (syringe pump; 750 μL min^-1^) of nuclease-free ultrapure water into the top of the tube. From each fraction, 60 μL was used for density measurement (Refractometer DR 301-95, Krüss) and weight determination (microbalance Satorius Extend) and the rest was precipitated with two volumes of ice-cold isopropanol (molecular range, >99%) and 4 μL glycogen (5 mg mL^-1^) for at least 30 min at -20°C. After precipitation, the fractions were centrifuged for 30 min at 4°C (139000 rpm) and the supernatant decanted. The residual pellet was washed with 500 μL 70% ethanol (molecular range, >99%) and centrifuged again as before. The supernatant was removed and the pellet resuspended in 30 μL nuclease-free ultrapure water. The RNA concentrations of all fractions were determined using the Quant-iT-RiboGreen RNA Assay Kit (Life Technologies).

For determining the ^13^C-enriched fractions in the CsTFA sample gradients, we aligned a ^13^C-enriched *Escherichia coli* gradient (“^13^C_acetate_ *E. coli* standard”, Supplementary Figure S4) and compared the locations of the ^13^C-peaks. To obtain the ^13^C_acetate_ *E. coli* standard, we incubated *E. coli* over 4 days in minimal medium (pH = 7) containing sodium hydrogen phosphate (Na_2_HPO_4_, 6 g L^-1^), potassium dihydrogen phosphate (KH_2_PO_4_, 3 g L^-1^), ammonium chloride (NH_4_Cl, 1 g L^-1^), sodium chloride (NaCL, 0.5 g L^-1^), magnesium sulfate (MgSO_4_, 0.12 g L^-1^), calcium chloride (CaCl_2_, 0.01 g L^-1^) and ^13^C-sodium acetate (^13^CH_3_^13^CO_2_Na, 99 atom-%, 0.25 g L^-1^) as sole carbon source. After incubation, RNA was extracted and purified, CsTFA density gradient centrifugation was carried out and RNA concentrations were quantified for 20 fractions as described for the sediment/slurry samples.

### Transcription, PCR Amplification, Terminal Restriction Fragment Length Polymorphism (T-RFLP) Analysis and Cloning

RNA of selected fractions was transcribed into complementary DNA (cDNA) using the Sensiscript RT Kit (Quiagen) with the primer U1492R (5′–GGYTACCTTGTTACGACTT–3′), following the manufacturer’s instructions.

For PCR amplification, for each sample a 50 μL PCR reaction mix containing ∼10 ng template cDNA, 20 pmol of each primer (A958R: 5′–YCCGGCGTTGAMTCCAATT–3′ and A20F 5′–TCYGGTTGATCCTGCCRG–3′), 1.25 U *Taq* polymerase (Fermentas), 40 nmol deoxynucleotides and 125 nmol MgCl_2_ in 1x *Taq* reaction buffer (Fermentas) was prepared on ice and cycled in the PCR cycler with the following program: 2 min at 95°C, followed by 39 cycles of 1 min at 95°C, 2 min at 55°C, 4 min at 72°C, 4 min and finally 10 min at 72°C.

For T-RFLP analyses, cDNA was amplified by PCR as described above with the primer pair A958R and A20F-FAM (5′–TCYGGTTGATCCTGCCRG–3′). Thereafter, 10 μL of each amplicon DNA sample was digested with 5 U *Bsu*RI (HaeIII) restriction enzyme (Fermentas) at 37°C for 8 h and thereafter purified with GeneJet PCR purification Kit (Fermentas Life Technologies). Terminal restriction fragment length polymorphism (T-RFLP) analysis was performed with an Applied Biosystems ABI3730XL sequencer (capillary electrophoresis) at Uppsala Genome Center (Sweden).

16S rRNA clone libraries were carried out for one representative gradient fraction of the ^13^C and ^12^C peak, each from the successful ^13^C_CH4_ and ^13^C_DIC_ incubation and from the T_0_ slurry and T_1_ core control. Hereby, 7 μL cDNA amplicon of each sample was blunted and ligated with a pJET 1.2/blunt Cloning vector using the CloneJET PCR Cloning Kit (Thermo-Scientific) and transformed into chemically competent *E. coli* cells (One Shot TOP 10F′ from Invitrogen) according to the manufacturer’s instructions. The cells were spread on pre-warmed LB plates containing ampicillin and incubated overnight at 37°C. Thereafter, clones were screened using a colony PCR with primers PJF (5′–CGACTCACTATAGGGAGAGCGGC–3′) and pJR (5′–AAGAACATCGATTTTCCATGGCAG–3′). The archaeal clones were sent to Macrogen Europe (Netherlands) for sequencing.

### Treatment of T-RFLP Data and Phylogenetic Analysis of Sequences

Accurate T-RF sizes were determined using Peak ScannerTM (Applied Biosystems) on the obtained T-RFLP data from Uppsala Genome Center. Thereafter, the online source T-RFLP analysis Expedited^1^ was used for noise filtration (noise filtration factor 1.0; peak area) and T-RF binning. All OTUs, which had a relative abundance < 1% were removed manually afterwards. Sequences obtained from the 16S rRNA libraries were scanned using the program “4 peaks” and low quality ends, vector and primer sequences were removed manually. Chimeras were detected by the online tool Bellerophon (Huber et al., 2004), evaluated manually and removed. Sequences, which passed the quality control, were imported into the SSU Reference 115 SILVA database for phylogenetic reconstructions (Pruesse et al., 2007) using the ARB software package (Ludwig et al., 2004). All 208 sequences have been deposited at GenBank under the accession numbers KX463063 – KX463269 (Supplementary Table S3).

## Results

### Sediment Biogeochemistry *In Situ* and in Incubations

**FIGURE 1:**
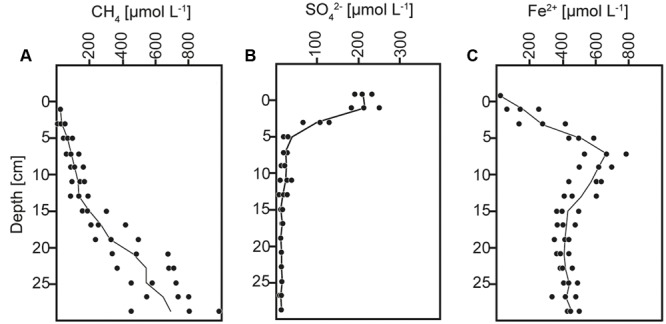
**Concentration depth profiles of (A)** methane (CH_4_), **(B)** sulfate (SO_4_^2-^), and **(C)** dissolved iron (Fe^2+^).

The anoxic sediment column contained sulfate until a depth of ∼15 cm with a maximum concentration of ∼200 μmol L^-1^ at the surface (**Figure 1B**), similar as previously reported (Norði et al., 2013; Weber et al., 2016). The methane content increased with depth and reached concentrations of ∼900 μmol L^-1^ (**Figure 1A**). AOM activity was indicated at 5–15 cm depth by the concave shape of the methane profile, and thereby occurred within the depth interval of sulfate consumption and iron reduction, as indicated by the distributions of sulfate and soluble Fe^2+^ (**Figure 1**), and consistent with previous direct rate measurements using radiotracer incubations and with analyses of particulate iron speciation (Norði et al., 2013; Weber et al., 2016).

**FIGURE 2:**
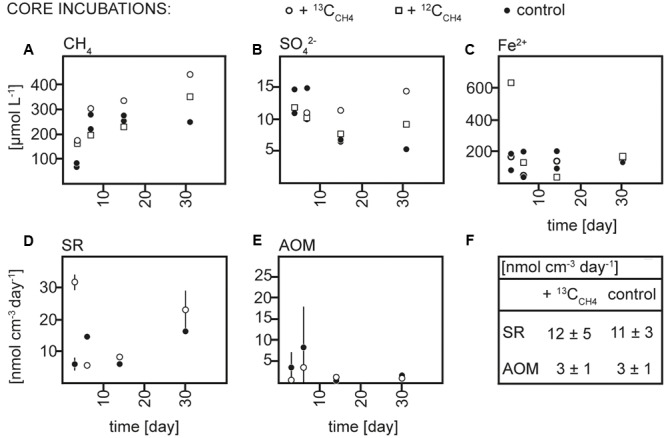
**Concentrations over time of (A)** methane (CH_4_), **(B)** sulfate (SO_4_^2-^), and **(C)** dissolved iron (Fe^2+^), rates over time of **(D)** sulfate reduction (SR) and **(E)** anaerobic methane oxidation (AOM) in ^13^C_CH4_, ^12^C_CH4_ and control core incubations and **(F)** average rates of SR and AOM in ^13^C_CH4_ and control core incubations.

**FIGURE 3:**
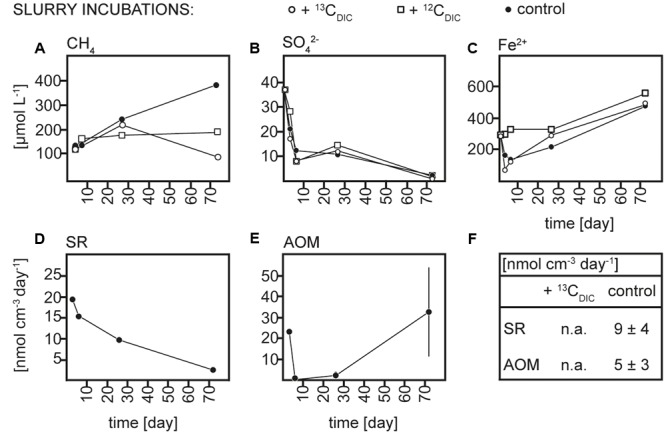
**Concentrations over time of (A)** methane (CH_4_), **(B)** sulfate (SO_4_^2-^), and **(C)** dissolved iron (Fe^2+^), rates over time of **(D)** sulfate reduction (SR) and **(E)** anaerobic methane oxidation (AOM) in ^13^C_DIC_, ^12^C_DIC_ and control slurry incubations and **(F)** average rates of SR and AOM in ^13^C_DIC_ and control slurry incubations; n.a., not analyzed.

Sediment and slurries were incubated with ^13^C_CH4_ or ^13^C_DIC_, their ^12^C analogs or without addition (controls) for maximum 30 and 72 days, respectively. As described in more detail below, ^13^C-labeling of RNA was achieved in the ^13^C_CH4_ core and ^13^C_DIC_ slurry incubations. We therefore focus on the biogeochemical results from these incubations here (**Figures 2, 3**), while the results of the ^13^C_DIC_ core and ^13^C_CH4_ slurry incubations are displayed in Supplementary Figures S1, S2. At incubation start, sulfate concentrations at 10–15 cm were higher in the slurries (∼37 μmol L^-1^) than in the cores (10–15 μmol L^-1^), due to the addition of sulfate-containing medium (**Figures 2B, 3B** and Supplementary Figures S1B, S2B). All slurry incubations showed a decrease in sulfate concentrations in all treatments with a mean rate of ∼0.3 ± 0.2 μmol L^-1^ d^-1^. In the core incubations, sulfate concentrations remained at 10–15 μmol L^-1^ or tended to decrease but only dropped below detection at the last time point in the ^12^C_DIC_ and ^13^C_DIC_ incubations. Methane concentrations increased in the control slurry incubation with ∼4 ± 3 μmol L^-1^ d^-1^, indicating net methanogenesis. A similar rate of increase in the control core incubation together with the decrease in sulfate indicated an upward shift of the redox zonation during the incubation while the increase in methane in the ^12^C_CH4_ and ^13^C_CH4_ cores and slurries reflected the repeated injection of methane to these. In the ^12^C_DIC_- and ^13^C_DIC_-amended core and slurry incubations, methane concentrations did not show a clear trend over time. Radiotracer incubations in control and ^13^C_CH4_ incubations confirmed SR and AOM activity in cores and slurries at all time points (**Figures 2D,E, 3D,E** and Supplementary Figures S2D,E; see also additional discussion in Supplementary). On average, SR showed rates of 11 ± 3 μmol L^-1^ d^-1^ and 12 ± 5 μmol L^-1^ d^-1^ in control and ^13^C_CH4_-amended cores, respectively, and average AOM rates of 3 ± 1 μmol L^-1^ d^-1^ in both (**Figures 2D,E**). In slurries, SR and AOM rates were similar to the rates in the core incubations, with an average SR rate of 9 ± 4 μmol L^-1^ d^-1^ in both control and ^13^C_CH4_-amended slurries and AOM rates of 5 ± 3 and 4 ± 2 μmol L^-1^ d^-1^ in control and ^13^C_CH4_-amended slurries, respectively (**Figures 3D,E**).

As the addition of ^13^C_DIC_, ^12^C_DIC_, ^13^C_CH4_ or ^12^C_CH4_ did not impact the biogeochemistry of the incubations nor SR and AOM rates substantially, we rule out a major impact on the microbial community due to amendments in comparison to the control incubations. Therefore, we interpret the control, ^12^C_CH4_ and ^13^C_CH4_ core incubations and the control, ^12^C_DIC_, ^13^C_DIC_ slurry incubations, respectively, as biogeochemical replicates.

### Taxonomic Identification of Archaea

The initial composition of the active part of the archaeal community was assessed by two 16S rRNA clone libraries originating from the slurry and from a core before ^13^C-addition. In total, 76 clones were retrieved, from which ∼40 % were assigned to the phylum *Euryarchaeota*, ∼8% to *Miscellaneous Euryarchaeotic Group* and *Aenigmarchaeota* and ∼52% to the phylum *Crenarchaeota* (**Table 1** and Supplementary Table S1). Within the *Euryarchaeota*, the two largest groups were *Thermoplasmatales* with 19 clones and the *Methanosarcinales* cluster AMNE-2d with nine clones, whereas *Methanosaeta* showed minor contributions. Almost all sequences that fell into the phylum *Crenarchaeota* were assigned to *Miscellaneous Crenarchaeotic Group*.

**Table 1:** Taxonomic affiliation and number of sequences obtained from 16S rRNA clone libraries at incubation start (T_0_) and for one representative sample of ^13^C_CH4_ and ^13^C_DIC_ incubations, each from the ^13^C fraction and the ^12^C fraction of the same CsTFA gradients.

Taxonomic affiliation	T_0_	^13^C_CH4_ core		^13^C_DIC_ slurry	
		^13^C	^12^C	^13^C	^12^C
***EURYARCHAEOTA***
*Thermoplasmata*
***Thermoplasmatales***	19		8	2	6
*Methanomicrobia*
*Methanosarcinales*
*Methanosaetacea*
***Methanosaeta***	3	3	1	3	
*Methanomicrobia*
*Methanosarcinales*
***ANME-2d***	9	22	1	17	
*Methanomicrobia*
*Methanomicrobiales*
***Methanoregulacea/Methanolinea***				2	
***MISCELLANEOUS***
***EURYARCHAEOTIC GROUP***	2		6		5
***AENIGMARCHAEOTA***	2		2		3
***CRENARCHAEOTA***
Marine Benthic Group B		1			
others	2		1		1
***MISCELLANEOUS***	39	1	21	5	21
***CRENARCHAEOTIC GROUP***

Sufficient amounts of ^13^C-labeled RNA for clear ^13^C- and ^12^C-fraction separation during CsTFA centrifugation and subsequent reverse transcription were obtained after 30 days in the ^13^C_CH4_ core incubation and after 73 days in the ^13^C_DIC_ slurry incubation, while no RNA was detected in, and no cDNA could be obtained by reverse transcription of the high density gradient fractions sampled earlier during the incubations. The cDNA generated from the ^12^C and ^13^C fractions of the density gradient was amplified and resulted in archaeal products that were used to construct 16S rRNA clone libraries and perform T-RFLP analysis.

**FIGURE 4:**
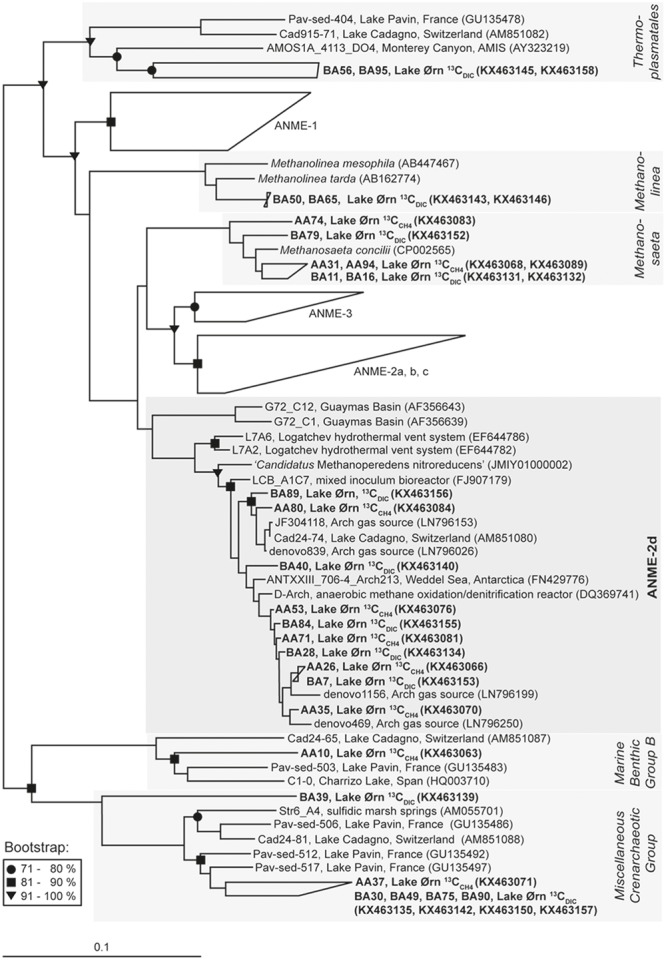
**Neighbor-joining 16S rRNA phylogenetic reconstruction for ^13^C-labeled sequences obtained from ^13^C_CH4_ and ^13^C_DIC_ RNA stable isotope probing incubations and nearest related phylogenetic groups with *Hydrogenobaculum acidophilum* as outgroup; in brackets: Gene Bank accession numbers.** For the cluster ANME-2d 10 of 39 sequences were chosen for display. Bootstrap values (%) represent the percentage of 1000 repetitions and scale bar shows 10 % distance.

The archaeal 16S rRNA clone library constructed from the ^13^C fraction of the ^13^C_CH4_ incubation was strongly enriched in sequences affiliated closely to ANME-2d, which made up 82% of the total (22 of 27; **Figure 4, Table 1** and Supplementary Table S1). Of the remainder, three sequences were closely related to the aceticlastic methanogen *Methanosaeta concilii* that also falls into *Methanosarcinales* (Barber et al., 2011), while one was affiliated to Marine Benthic Group B in the phylum *Crenarchaeota* and was most closely related to clones from other lakes including AOM-active sediment from Lake Cadagno in Switzerland (Schubert et al., 2011). Most ANME-2d clones fell within a single OTU (Supplementary Table S1) and all were also closely related to AAA sequences retrieved from Lake Cadagno sediment (Schubert et al., 2011), sequences retrieved from a freshwater gas source (Timmers et al., 2016) and from the AOM zone of Antarctic cold seep sediments (Niemann et al., 2009). Furthermore, Lake Ørn ANME-2d sequences clustered with sequences derived from denitrifying methane-oxidizing bioreactors (Raghoebarsing et al., 2006; Hu et al., 2009) and with the freshwater nitrate-reducing anaerobic methane oxidizer ‘*Candidatus* Methanoperedens nitroreducens’ (Haroon et al., 2013).

A 16S rRNA clone library from the ^13^C fraction of the ^13^C_DIC_ slurry incubation yielded 29 sequences of which ∼83% fell into the phylum *Euryarchaeota* and ∼17% fell into the phylum *Crenarchaeota* (**Figure 4, Table 1** and Supplementary Table S1). The majority of the euryarchaeotic sequences again fell into *Methanosarcinales* and therein mainly into the group ANME-2d, together with the ANME-2d sequences retrieved from the ^13^C peak of the ^13^C_CH4_ incubation, as described above. A smaller amount of sequences from the ^13^C peak with ^13^C_DIC_ fell into the genus *Methanosaeta* and clustered together with the sequences from heavy fraction of the ^13^C_CH4_ incubation and the aceticlastic methanogen *Methanosaeta concilii* (Barber et al., 2011). Furthermore, a few sequences of the ^13^C fraction of the ^13^C_DIC_ slurry incubation affiliated with *Thermoplasmatales* and the methanogenic genus *Methanolinea.* All ^13^C-labeled crenarchaeotic sequences from the ^13^C_DIC_ incubation fell into *Miscellaneous Crenarchaeotic Group*.

Comparison of 16S rRNA from the ^13^C peak fractions to that from the ^12^C peak of the ^13^C_CH4_ and ^13^C_DIC_ incubations further confirmed the selective ^13^C labeling of the cluster ANME-2d. The ^12^C-peak clone libraries contained 40 and 36 clones from the ^13^C_CH4_ and ^13^C_DIC_ incubation, respectively. Similar to the initial communities, about half of the total sequences clustered in the *Miscellaneous Crenarchaeotic Group*. In total ∼20% of the sequences clustered in *Euryarchaeota* and were assigned to *Thermoplasmatales*, except one sequence that was assigned to *Methanosaeta* and one sequence that was assigned to ANME-2d in the ^13^C_CH4_ incubation. The rest of the sequences were assigned to *Miscellaneous Euryarchaeotic Group* and *Aenigmarchaeota*.

Transcription, PCR amplification, and terminal restriction fragment length polymorphism analyses were carried out for the same samples that were used to construct 16S rRNA clone libraries with the aim to obtain a representation of the entire archaeal community (Supplementary Figure S3). Overall, a large part of the archaeal community could be taxonomically identified based on the predicted T-RF length both for incubation start and after incubation with the ^13^C-labeled substrates. At incubation start, 69 and 77% of the OTUs in core and slurry, respectively, could be aligned with the OTUs obtained from the 16S rRNA clone libraries. In the ^13^C_CH4_ core incubation, ∼74% of the OTUs retrieved from the ^13^C peak and in the ^13^C_DIC_ slurry incubation ∼69% of the OTUs retrieved from the ^13^C peak could be taxonomically identified. The Supplementary section provides a detailed discussion about the T-RFLP data (Supplementary Table S2) and an overview over the archaea identified in Lake Ørn sediment in the present study.

## Discussion

### Methane and Sulfur Turnover *In Situ* and in Incubations

Anaerobic consumption of methane in Lake Ørn sediment was indicated in the depth interval of 5–15 cm by the concave increase of methane concentrations with depth and confirmed by radiotracer incubations and stable isotope analyses in previous reports (Norði et al., 2013; Weber et al., 2016). Nitrate can be excluded as electron acceptor for AOM as it was depleted within the upper 2 cm of the sediment (Norði et al., 2013). As potential electron acceptors for AOM in Lake Ørn, both sulfate and poorly crystalline ferric iron were previously discussed (Norði et al., 2013; Weber et al., 2016). However, elevated sulfate reduction rates in the AOM zone and a methane-dependent alteration of δ^34^S signatures in slurry incubations directly implicated sulfate as electron acceptor for AOM while a coupling to iron might be indirect via an iron(III)-dependent cryptic sulfur cycle (Weber et al., 2016).

We chose to focus on the depth interval with the highest *in situ* AOM activities (10–15 cm, **Figures 2E, 3E**; see also Norði et al., 2013; Weber et al., 2016) in both cores and slurries with the aim of exploiting the different advantages of each of these two incubation types. Generally, in core incubations, the microbial communities are maintained in the natural biogeochemical gradients and largely protected from mechanical disturbances. However, the quantification of biogeochemical processes in a defined interval is rather difficult as porewater and gaseous parameters are strongly impacted by diffusion along concentration gradients, and core-to-core variability adds noise to the temporal changes observed. Higher controllability can be achieved in slurry incubations where the addition of medium can also support the enrichment of a preferred part of the microbial community. Disadvantages of slurring are physical disturbances of the microbial community and the blockage of diffusion of products and substrates such as, e.g., sulfate due to the closed system.

Anaerobic oxidation of methane and SR were active in all incubations and their rates as obtained from radiotracer incubations were comparable between cores and slurries (**Figures 2D,E, 3D,E**). The net consumption of sulfate in all slurry incubations and an overall tendency of sulfate to decrease in the core incubations further confirmed microbial reduction of sulfate. Still, radiotracer-based rates exceeded the rate of sulfate depletion substantially, indicating active reoxidation of sulfide as previously discussed for this site (Norði et al., 2013; Weber et al., 2016). Similarly, a net accumulation of methane occurred in most incubations and suggested a co-occurrence of AOM and methanogenesis in the layer of sulfate depletion. Based on the similarity to the previous analyses of AOM in Lake Ørn we conclude that AOM in our incubations was likely coupled to sulfate reduction, at least in part, but also that a direct coupling to iron reduction cannot be excluded.

### ANME-2d Assimilated ^13^C_CH4_-derived ^13^C

In the sediment core incubations, ^13^C was assimilated into 16S rRNA after ^13^C_CH4_ addition, giving rise to a high-density peak in the density gradient, while radiotracer incubations confirmed AOM activity. Far most of the ^13^C-labeled sequences fell into the euryarchaeotic order *Methanosarcinales* and therein grouped tightly in the cluster ANME-2d (**Figure 4** and **Table 1**). Sequences falling into ANME-2d were also part of the active initial archaeal community, 12% of clones, whereas strains from other ANME groups were not detected. The presence of ANME-2d in the 16S rRNA clone libraries of the untreated sediment and the strong enrichment of this group in the high-density fraction obtained from ^13^C labeling, indicated that ANME-2d carry out AOM in Lake Ørn sediment. This was further supported by the lack of detectable RNA in the high-density fractions of the parallel incubation without ^13^C source (Lueders et al., 2004).

Members of this cluster were previously observed in AOM-active habitats, but due to lacking evidence of their methane-oxidizing capabilities, they were first grouped with uncultured methanogens in a clade named GOM Arc 1 (Lloyd et al., 2006). Subsequently, the identification of 16S rRNA sequences associated with nitrate-dependent AOM archaea in a bioreactor enrichment (Raghoebarsing et al., 2006) resulted in identification of the cluster named AOM-Associated Archaea (AAA) as a subgroup of GOM ARC 1 (Knittel and Boetius, 2009). The group AAA was renamed ANME-2d after the genetic reconstruction of ‘*Cand.* M. nitroreducens’, with the capability to reduce nitrate while oxidizing methane via the reverse methanogenesis pathway (Haroon et al., 2013).

In Lake Ørn, nitrate can be excluded as electron acceptor for AOM (Norði et al., 2013), suggesting that the detected ANME-2d strains most likely coupled methane oxidation to sulfate and/or iron reduction. Ferric iron-dependent AOM was recently reported in an enriched ANME-2d/AAA strain (Ettwig et al., 2016). However, a coupling of ANME-2d to sulfur cycling cannot be conclusively excluded as this group was also detected in a range of marine sulfur-cycling environments including, e.g., the Logatchev hydrothermal vent system (Voordeckers et al., 2008), deep-sea marine sediments (e.g., Inagaki et al., 2006), and Antarctic cold seep sediments (Niemann et al., 2009). Further ANME-2d sequences were reported from freshwater systems characterized by active sulfur-cycling at lower sulfate concentrations as, e.g., in sediment of the alpine Lake Cadagno (originally described as AAA; Schubert et al., 2011), in freshwater wells (Flynn et al., 2013), and in freshwater gas sources (Timmers et al., 2016).

Although we cannot link the members of ANME-2d identified in Lake Ørn to either sulfate or ferric iron reduction with certainty, the present study is, to our knowledge, the first to experimentally associate ANME-2d activity to AOM in a natural environment by following the incorporation of ^13^C_CH4_ into biomass, and to demonstrate that this activity is possible in the absence of nitrate.

In the ^13^C_CH4_ incubation, a small number of sequences clustering with *Methanosaeta, Marine Benthic Group*, and *Miscellaneous Crenarchaeotic Group* were detected in the high-density fraction (**Figure 4, Table 1** and Supplementary Table S1). As these groups were not clearly enriched in the high-density fraction relative to the original community, they may represent the carryover of small amounts of unlabeled RNA to the ^13^C peak, which is typical for the method and possibly due to interaction with ^13^C-labeled RNA (Lueders et al., 2004). The same consideration applies to the minor groups detected in the heavy fraction of the ^13^C_DIC_ incubation (**Figure 4, Table 1** and Supplementary Table S1). Like previous RNA-SIP studies (Hori et al., 2007; Gutierrez et al., 2015), we therefore exclude these minor groups from both treatments from further discussion.

### Incorporation of ^13^C_CH4_- and ^13^C_DIC_-derived ^13^C into ANME-2d Strains: Mixotrophy or Cross-feeding?

Reverse methanogenesis as the pathway for anaerobic methane oxidation in ANME strains including ANME-2d is known on a detailed enzymatic level (Hallam et al., 2004; Haroon et al., 2013; Arshad et al., 2015). Furthermore, as shown in the present study, members of the ANME-2d cluster can assimilate CH_4_ and incorporate it into biomass. However, from our ^13^C_DIC_ slurry, we obtained ^13^C-enriched 16S rRNA sequences affiliated to ANME-2d and therein clustering with the sequences obtained in the ^13^C_CH4_ core incubation. This finding suggests that ANME-2d strains are either capable of a mixed assimilation of CH_4_ and DIC and/or that cross-feeding of ^13^C-labeled metabolites could potentially have occurred.

Cross-feeding of labeled substrates is a general risk in stable isotope probing incubations (Dumont and Murrell, 2005 and references therein). In the present study, cross-feeding could potentially have occurred in two directions: (1) in the ^13^C_CH4_ incubations, AOM activity could have lead to the build-up of ^13^C_DIC_, which could have been assimilated by autotrophic microorganisms, e.g., by hydrogenotrophic methanogens, being falsely understood as methanotrophs. On the other hand, (2) in the ^13^C_DIC_ incubations, methanogens could have built up significant amounts of ^13^C_CH4_ and ^13^C_CH4_-assimilating anaerobic methane oxidizers could have been falsely interpreted as autotrophs. We exclude case (1) because the highest accumulation of ^13^C in the DIC pool achieved during the incubation would be insufficient to explain our observations. Thus, with an average AOM rate of 3 nmol cm^-3^ day^-1^ in the 10–15 cm layer of the ^13^C_CH4_ incubated core (**Figure 2**), over the incubation period of 30 days, the amount of ^13^C_DIC_ generated by AOM would have reached only ∼3 atom-% of the total DIC pool (∼3 mmol L^-1^, data not shown) at the end of incubation, while 20 atom-% labeling is generally needed to shift a RNA sequence from the ^12^C to the ^13^C fraction in the CsTFA gradient (Manefield et al., 2011). A similar estimate can be performed to evaluate (2) cross-feeding in the ^13^C_DIC_ incubations: as there was no significant net change in methane concentrations in the ^13^C_DIC_ slurry incubations, one can assume that methanogenic activity may have approximately equalled the activity of anaerobic methanotrophs. As a consequence, over an incubation time of 72 days, with an average rate of 5 nmol cm^-3^ day^-1^ and the DIC pool being ^13^C enriched by ∼25%, methanogens could have built up >100 μmol L^-1^ of ^13^C_CH4_ which would have been sufficient for cross-feeding under the prevailing methane conditions. Therefore, it appears evident that ANME-2d in Lake Ørn directly assimilated ^13^C_CH4_, but the apparent ^13^C_DIC_ assimilation by the same strain may be a result of ^13^CH_4_ incorporation after methanogenesis from ^13^C_DIC_.

Mixed assimilation of CH_4_ and CO_2_ has been reported for ANME-2a and -2c strains retrieved from marine environments, but with a preferred usage of CO_2_ over CH_4_ for biomass production (Wegener et al., 2008; Milucka et al., 2012). Thus, ANME-2 strains of a long-term enrichment culture incorporated both CO_2_ and CH_4_ into their biomass, but with a 25-times higher rate for CO_2_ in comparison to CH_4_ (Milucka et al., 2012). A complete decoupling of methane oxidation from the assimilatory system and autotrophy instead of mixotrophy was proposed for a group of ANME-1 strains from hydrothermal sediments in Guaymas Basin (Kellermann et al., 2012). It remains unclear if the preference of the carbon source for assimilation is strain-specific or/and impacted by environmental conditions. Our observation of incorporation of ^13^C into RNA of ANME-2d in both ^13^C_CH4_ and ^13^C_DIC_ incubations suggests that carbon flow in the ANME-2d cells may differ from that in the previously studied ANME-1, -2a, and -2c strains. However, the difference in labeling pattern between cores and slurries might further indicate a metabolic shift of the AOM-mediating community caused by the different types of incubation, as the ^13^C sources were added in comparable amounts in slurry and core incubations and samples were treated consistently. The previous studies were all carried out in slurry incubations or enrichment cultures (Wegener et al., 2008; Kellermann et al., 2012; Milucka et al., 2012). Their results were in agreement with the results of our slurry incubations that only showed ^13^C-enriched RNA with ^13^C_DIC_-amendment and therefore also indicated preferred assimilation of CO_2_. We conclude that, members of ANME-2d may have the potential for mixed assimilation of CH_4_ and CO_2_, but further investigations are required to identify if such a metabolism would be strain-specific or a physiological response to changing biogeochemical environments.

## Conclusion

In freshwater environments, AOM was only recently shown to substantially reduce methane emissions from sediment and prevents its flux into the atmosphere (Norði et al., 2013; Segarra et al., 2013; Deutzmann et al., 2014). In Lake Ørn, AOM was active below the nitrate-containing zone and under low-sulfate, iron-rich conditions (Norði et al., 2013; Weber et al., 2016) and carried out by anaerobic methane oxidizers belonging to the cluster ANME-2d. We showed that members of ANME-2d are active under natural conditions and own the enzymatic capabilities to assimilate CH_4_ and probably also CO_2_ into their biomass. Our results place members ANME-2d as prime candidates for AOM in low-sulfate iron-rich environments, and thereby as potentially important players in the regulation of methane emissions from freshwater systems. Further studies of this clade in Lake Ørn and other freshwater environments might help to solve the puzzle of the pathways involved in anaerobic methanotrophy in such systems.

## Author Contributions

HW, KH, and BT designed research, analyzed and interpreted the data, and wrote the manuscript. HW carried out field work and laboratory-based research.

## Conflict of Interest Statement

The authors declare that the research was conducted in the absence of any commercial or financial relationships that could be construed as a potential conflict of interest.

## Footnotes

1 http://trex.biohpc.org/

## References

[B1] Arshad A. Speth D. R. De Graaf R. M. Op den Camp H. J. M. Jetten M. S. Welte C. U. (2015). A metagenomics-based metabolic model of nitrate-dependent anaerobic oxidation of methane by *Methanoperedens*-like archaea. *Front. Microbiol.* 6:1423 10.3389/fmicb.2015.01423 PMC4683180 26733968

[B2] Barber R. D. Zhang L. Harnack M. Olson M. V. Kaul R. Ingram-Smith C. (2011). Complete genome sequence of *Methanosaeta concilii*, a specialist in aceticlastic methanogenesis. *J. Bacteriol.* 193 3668–3669. 10.1128/JB.05031-11 21571998 PMC3133334

[B3] Beal E. J. Claire M. W. House C. H. (2011). High rates of anaerobic methanotrophy at low sulfate concentrations with implications for past and present methane levels. *Geobiology* 9 131–139. 10.1111/j.1472-4669.2010.00267.x 21231994

[B4] Beal E. J. House C. H. Orphan V. J. (2009). Manganese- and iron-dependent marine methane oxidation. *Science* 325 184–187. 10.1126/science.1169984 19589998

[B5] Boetius A. Ravenschlag K. Schubert C. J. Rickert D. Widdel F. Gieseke A. (2000). A marine microbial consortium apparently mediating anaerobic oxidation of methane. *Nature* 407 623–626. 10.1038/35036572 11034209

[B6] De Brabandere L. Thamdrup B. Revsbech N. P. Foadi R. (2012). A critical assessment of the occurrence and extend of oxygen contamination during anaerobic incubations utilizing commercially available vials. *J. Microbiol. Methods* 88 147–154. 10.1016/j.mimet.2011.11.001 22101311

[B7] Deutzmann J. S. Stief P. Brandes J. Schink B. (2014). Anaerobic methane oxidation coupled to denitrification is the dominant methane sink in a deep lake. *Proc. Natl. Acad. Sci. U.S.A.* 111 18273–18278. 10.1073/pnas.1411617111 25472842 PMC4280587

[B8] Dumont M. G. Murrell J. C. (2005). Stable isotope probing – linking microbial identity to function. *Nat. Rev. Microbiol.* 3 499–504. 10.1038/nrmicro1162 15886694

[B9] Egger M. Rasigraf O. Sapart C. J. Jilbert T. Jetten M. S. Röckmann T. (2015). Iron-mediated anaerobic oxidation of methane in brackish coastal sediments. *Environ. Sci. Technol.* 49 277–283. 10.1021/es503663z 25412274

[B10] Ettwig K. F. Butler M. K. Le Paslier D. Pelletier E. Mangenot S. Kuypers M. M. M. (2010). Nitrite-driven anaerobic methane oxidation by oxygenic bacteria. *Nature* 464 543–548. 10.1038/nature08883 20336137

[B11] Ettwig K. F. Zhu B. Speth D. Keltjens J. T. Jetten M. S. M. Kartal B. (2016). Archaea catalyze iron-dependent anaerobic oxidation of methane. *Proc. Natl. Acad. Sci. U.S.A.* 10.1073/pnas.1609534113 [Epub ahead of print]. PMC5111651 27791118

[B12] Flynn T. M. Sanford R. A. Ryu H. Bethke C. M. Levine A. D. Ashbolt N. J. (2013). Functional microbial diversity explains groundwater chemistry in a pristine aquifer. *BMC Microbiol.* 13 1–15. 10.1186/1471-2180-13-146 23800252 PMC3700874

[B13] Gutierrez T. Biddle J. F. Teske A. Aitken M. D. (2015). Cultivation-dependent and cultivation-independent characterization of hydrocarbon-degrading bacteria in Guaymas Basin sediments. *Front. Microbiol.* 6:695 10.3389/fmicb.2015.00695 PMC4493657 26217326

[B14] Hallam S. J. Putnam N. Preston C. M. Detter J. C. Rokhsar D. Richardson P. M. (2004). Reverse methanogenesis: testing the hypothesis with environmental genomics. *Science* 305 1457–1462. 10.1126/science.1100025 15353801

[B15] Haroon M. F. Hu S. Shi Y. Imelfort M. Keller J. Hugenholtz P. (2013). Anaerobic oxidation of methane coupled to nitrate reduction in a novel archaeal lineage. *Nature* 500 567–570. 10.1038/nature12375 23892779

[B16] Hinrichs K.-U. Hayes J. M. Sylva S. P. Brewer P. G. DeLong E. F. (1999). Methane-consuming archaebacteria in marine sediments. *Nature* 398 802–805. 10.1038/19751 10235261

[B17] Holmkvist L. Ferdelman T. G. Jørgensen B. B. (2011). A cryptic sulfur cycle driven by iron in the methane zone of marine sediment (Aarhus Bay, Denmark). *Geochim. Cosmochim. Acta* 75 3581–3599. 10.1016/j.gca.2011.03.033

[B18] Hori T. Noll M. Igarashi Y. Friedrich M. W. Conrad R. (2007). Identification of acetate-assimilating microorganisms under methanogenic conditions in anoxic rice field soil by comparative stable isotope probing of RNA. *Appl. Environ. Microbiol.* 73 101–109. 10.1128/AEM.01676-06 17071795 PMC1797110

[B19] Hu S. Zeng R. J. Burow L. C. Lant P. Keller J. Yuan Z. (2009). Enrichment of denitrifying anaerobic methane oxidizing microorganisms. *Environ. Microbiol. Rep.* 1 377–384. 10.1111/j.1758-2229.2009.00083.x 23765890

[B20] Huber T. Faulkner G. Hugenholtz P. (2004). Bellerophon: a program to detect chimeric sequences in multiple sequence alignments. *Bioinformatics* 20 2317–2319. 10.1093/bioinformatics/bth226 15073015

[B21] Inagaki F. Nunoura T. Nakagawa S. Teske A. Lever M. Lauer A. (2006). Biogeographical distribution and diversity of microbes in methane hydrate-bearing deep marine sediments on the Pacific Ocean Margin. *Proc. Natl. Acad. Sci. U.S.A.* 103 2815–2820. 10.1073/pnas.0511033103 16477011 PMC1413818

[B22] Jørgensen B. B. (1978). Comparison of methods for quantification of bacterial sulfate reduction in coastal marine sediments. 1. Measurement with radiotracer techniques. *Geomicrobiol. J.* 1 11–27.

[B23] Kallmeyer J. Ferdelman T. G. Weber A. Fossing H. Jørgensen B. B. (2004). A cold chromium distillation procedure for radiolabeled sulfide applied to sulfate reduction measurements. *Limnol. Oceanogr. Methods* 2 171–180. 10.4319/lom.2004.2.171

[B24] Kellermann M. Y. Wegener G. Elvert M. Yoshinaga M. Y. Lin Y.-S. Holler T. (2012). Autotrophy as a predominant mode of carbon fixation in anaerobic methane-oxidizing microbial communities. *Proc. Natl. Acad. Sci. U.S.A.* 109 19321–19326. 10.1073/pnas.1208795109 23129626 PMC3511159

[B25] Knittel K. Boetius A. (2009). Anaerobic oxidation of methane: progress with an unknown process. *Annu. Rev. Microbiol.* 63 311–334. 10.1146/annurev.micro.61.080706.093130 19575572

[B26] Lloyd K. G. Lapham L. Teske A. (2006). An anaerobic methane-oxidizing community of ANME-1b archaea in hypersaline Gulf of Mexico sediments. *Appl. Environ. Microbiol.* 72 7218–7230. 10.1128/AEM.00886-06 16980428 PMC1636178

[B27] Ludwig W. Strunk O. Westram R. Richter L. Meier H. Yadhukumar (2004). ARB: a software environment for sequence data. *Nucleic Acids Res.* 32 1363–1371. 10.1093/nar/gkh293 14985472 PMC390282

[B28] Lueders T. Manefield M. Friedrich M. W. (2004). Enhanced sensitivity of DNA- and rRNA-based stable isotope probing by fractionation and quantitative analysis of isopycnic centrifugation gradients. *Environ. Microbiol.* 6 73–78. 10.1046/j.1462-2920.2003.00536.x 14686943

[B29] Manefield M. Gutierrez-Zamora M.-L. Whiteley A. S. (2011). “RNA stable isotope probing,” in *Stable Isotope Probing and Related Technologies* eds Murrell J. C. Whiteley A. S. (Washington, DC: ASM Press) 25–36.

[B30] McGlynn S. E. Chadwick G. L. Kempes C. P. Orphan V. J. (2015). Single cell activity reveals direct electron transfer in methanotrophic consortia. *Nature* 526 531–535. 10.1038/nature15512 26375009

[B31] Meulepas R. J. W. Jagersma C. G. Khadem A. F. Stams A. J. M. Lens P. N. L. (2010). Effect of methanogenic substrates on anaerobic oxidation of methane and sulfate reduction by an anaerobic methanotrophic enrichment. *Appl. Microbiol. Biotechnol.* 87 1499–1506. 10.1007/s00253-010-2597-0 20445975 PMC2892604

[B32] Milucka J. Ferdelman T. G. Polerecky L. Franzke D. Wegener G. Schmid M. (2012). Zero-valent sulphur is a key intermediate in marine methane oxidation. *Nature* 491 541–546. 10.1038/nature11656 23135396

[B33] Niemann H. Fischer D. Graffe D. Knittel K. Montiel A. Heilmayer O. (2009). Biogeochemistry of a low-activity cold seep in the Larsen B area, western Weddell Sea, Antarctica. *Biogeosciences* 6 2383–2395. 10.5194/bg-6-2383-2009

[B34] Norði K. Á. Thamdrup B. Schubert C. J. (2013). Anaerobic oxidation of methane in an iron-rich Danish freshwater lake sediment. *Limnol. Oceanogr.* 58 546–554. 10.4319/lo.2013.58.2.0546

[B35] Oni O. Miyatake T. Kasten S. Richter-Heitmann T. Fischer D. Wagenknecht L. (2015). Distinct microbial populations are tightly linked to the profile of dissolved iron in the methanic sediments of the Helgoland mud area, North Sea. *Front. Microbiol.* 6:365 10.3389/fmicb.2015.00365 PMC4416451 25983723

[B36] Orphan V. J. House C. H. Hinrichs K.-U. McKeegan K. D. DeLong E. F. (2002). Multiple archaeal groups mediate methane oxidation in anoxic cold seep sediments. *Proc. Natl. Acad. Sci. U.S.A.* 99 7663–7668. 10.1073/pnas.072210299 12032340 PMC124316

[B37] Pruesse E. Quast C. Knittel K. Fuchs B. M. Ludwig W. Glöckner F. O. (2007). SILVA: a comprehensive online resource for quality checked and aligned ribosomal RNA sequence data compatible with ARB. *Nucleic Acids Res.* 35 7188–7196. 10.1093/nar/gkm864 17947321 PMC2175337

[B38] Raghoebarsing A. A. Pol A. Van de Pas-Schoonen K. T. Smolders A. J. P. Ettwig K. F. Rijpstra W. I. C. (2006). A microbial consortium couples anaerobic methane oxidation to denitrification. *Nature* 440 918–921. 10.1038/nature04617 16612380

[B39] Riedinger N. Brunner B. Formolo M. J. Solomon E. Kasten S. Strasser M. (2010). Oxidative sulfur cycling in the deep biosphere of the Nankai Trough, Japan. *Geology* 38 851–854. 10.1130/G31085.1

[B40] Riedinger N. Formolo M. J. Lyons T. W. Henkel S. Beck A. Kasten S. (2014). An inorganic geochemical argument for coupled anaerobic oxidation of methane and iron reduction in marine sediments. *Geobiology* 12 172–181. 10.1111/gbi.12077 24460948

[B41] Røy H. Weber H. S. Tarpgaard I. H. Ferdelman T. G. Jørgensen B. B. (2014). Determination of dissimilatory sulfate reduction rates in marine sediment via radioactive 35S tracer. *Limnol. Oceanogr. Methods* 12 196–211. 10.4319/lom.2014.12.196

[B42] Scheller S. Yu H. Chadwick G. L. McGlynn S. E. Orphan V. J. (2016). Artificial electron acceptors decouple archaeal methane oxidation from sulfate reduction. *Science* 351 703–707. 10.1126/science.aad7154 26912857

[B43] Schubert C. J. Vazquez F. Lösekann-Behrens T. Knittel K. Tonolla M. Boetius A. (2011). Evidence for anaerobic oxidation of methane in sediments of a freshwater system (Lago Di Cadagno). *FEMS Microbiol. Ecol.* 76 26–38. 10.1111/j.1574-6941.2010.01036.x 21244447

[B44] Segarra K. E. A. Comerford C. Slaughter J. Joye S. B. (2013). Impact of electron acceptor availability on the anaerobic oxidation of methane in coastal freshwater and brackish wetland sediments. *Geochim. Cosmochim. Acta* 115 15–30. 10.1016/j.gca.2013.03.029

[B45] Sivan O. Adler M. Pearson A. Gelman F. Bar-Or I. John S. G. (2011). Geochemical evidence for iron-mediated anaerobic oxidation of methane. *Limnol. Oceanogr.* 56 1536–1544. 10.4319/lo.2011.56.4.1536

[B46] Skovgaard H. (2004). “*Ørn Sø 2003*”. Environment Monitoring Report, County of Aarhus Department of Nature and Environment, Højbjerg 63.

[B47] Timmers P. H. Suarez-Zuluaga D. A. Van Rossem M. Diender M. Stams A. J. Plugge C. M. (2016). Anaerobic oxidation of methane associated with sulfate reduction in a natural freshwater gas source. *ISME J.* 10 1400–1412. 10.1038/ismej.2015.213 26636551 PMC5029187

[B48] Treude T. Krüger M. Boetius A. Jørgensen B. B. (2005). Environmental control on anaerobic oxidation of methane in the gassy sediments of Eckernförde Bay (German Baltic). *Limnol. Oceanogr.* 50 1771–1786. 10.4319/lo.2005.50.6.1771

[B49] Voordeckers J. W. Do M. H. Hügler M. Ko V. Sievert S. M. Vetriani C. (2008). Culture dependent and independent analyses of 16S rRNA and ATP citrate lyase genes: a comparison of microbial communities from different black smoker chimneys on the Mid-Atlantic Ridge. *Extremophiles* 12 627–640. 10.1007/s00792-008-0167-5 18523725

[B50] Weber H. S. Thamdrup B. Habicht K. S. (2016). High sulfur isotope fractionation associated with anaerobic oxidation of methane in a low-sulfate, iron-rich environment. *Front. Earth Sci.* 4:61 10.3389/feart.2016.00061

[B51] Wegener G. Krukenberg V. Riedel D. Tegetmeyer H. E. Boetius A. (2015). Intercellular wiring enables electron transfer between methanotrophic archaea and bacteria. *Nature* 526 587–589. 10.1038/nature15733 26490622

[B52] Wegener G. Niemann H. Elvert M. Hinrichs K.-U. Boetius A. (2008). Assimilation of methane and inorganic carbon by microbial communities mediating the anaerobic oxidation of methane. *Environ. Microbiol.* 10 2287–2298. 10.1111/j.1462-2920.2008.01653.x 18498367

[B53] Whiteley A. S. Thomson B. Lueders T. Manefield M. (2007). RNA stable-isotope probing. *Nat. Protoc.* 2 838–844. 10.1038/nprot.2007.115 17446884

