# Supplementary material for: Anaerobic Methanotrophic Archaea of the ANME-2d Cluster Are Active in a Low-sulfate, Iron-rich Freshwater Sediment
Source: Front Microbiol. 2017 Apr 12;8:619. doi: 10.3389/fmicb.2017.00619 (PMC5389135; doi:10.3389/fmicb.2017.00619)
Supplement: Supplementary file 1 [file Table_1.pdf]

Supplementary:

Anaerobic methanotrophic archaea of the ANME-2d cluster are active in a low-sulfate, iron-rich freshwater sediment

Hannah Sophia Weber[1*], Kirsten Silvia Habicht[1,2], Bo Thamdrup[1]

[1]Nordic Center for Earth Evolution and Department of Biology, University of Southern Denmark, Odense, Dk

[2]Current address: Unisense A/S, Århus, Dk

***Corresponding author:** Hannah Sophia Weber (Hannah@biology.sdu.dk)

1 SR and AOM rates

In both control and $^{13}CH_4$- amended core incubations, sulfate reduction rates ranged from ~6 ± 0.1 to 32 ± 2 nmol cm^{-3} day^{-1} without showing a trend over time (Figure 2D). In the same cores, AOM rates were in the range of 1 ± 0.1 to 9 ± 8 nmol cm^{-3} day^{-1}, whereas highest rates were measured in the first two time points and thus, AOM activity apparently decreased with time (Figure 2E). In slurry incubations, SR rates decreased over time both in control and $^{13}CH_4$-amended incubations from 19 ± 0.6 nmol cm^{-3} day^{-1} to 1 ± 0 nmol cm^{-3} day^{-1} (Figure 3D). AOM rates increased over time from the detection limit, reaching up to 33 ± 21 nmol cm^{-3} day^{-1} at day 72 (Figure 3E), and clearly exceeded SR rates at this last time point of the incubation.

Lake Ørn sediment is characterized by rapid iron-driven reoxidation processes and cryptic sulfur cycling involving microbial disproportionation of intermediate sulfur species (Norði *et al.*, 2013, Weber et al., 2016). Therefore, microbially produced ^{35}S sulfides were most likely partially reoxidized and SR rates may be underestimated several fold, thus reflecting net instead of gross rates (Moeslund et al., 1994). At the same time, a previous Lake Ørn study (Norði et al., 2013) proposed that methanogenesis was co-occurring with AOM in the AOM zone, which was also indicated in our incubations by relatively constant methane concentrations despite AOM activity and a slight net methane accumulation in the control incubations. Therefore, even though $^{13}C_{DIC}$ produced by anaerobic methane oxidizers would have been diluted in a large DIC pool and consequently, the error should be relatively small, some of it could have been microbially re-converted to $^{13}CH_4$, causing an underestimation of AOM rates.

Therefore, SR and AOM rates presented here serve to confirm the activity of microbial sulfate reducers and anaerobic methane oxidizers. However, the magnitude of the rates as well as the corresponding SR:AOM ratio may be interpreted with caution.

2 Assessment of archaeal community by T-RFLP analyses

T-RFLP analyses were carried out for the same samples that were used to construct the 16S rRNA clone libraries with the aim to map the entire archaeal community. Operational taxonomic units (OTUs) obtained from T-RFLP analyses were then compared with true OTUs calculated from sequences from the 16S rRNA clone

libraries to gain an overview of the proportion of the taxonomically identified versus unidentified part of the archaeal community. A T-RF drift was observed between true T-RF lengths and observed T-RF lengths as described previously (Kaplan and Kitts, 2003), which resulted in this study in the addition of +1 bp between T-RFs obtained from 16S rRNA clone library derived T-RFs and observed T-RF lengths.

At incubation start, in slurry and core samples from layer 10 - 15 cm, the OTUs 93, 212 and 213 were strongly dominating in the T-RFLP results (Figures S3, Tables S1, S2). In the rRNA clone libraries, OTU 94 could be assigned to *Miscellaneous Crenarchaeotic Group.* The 16S rRNA clone libraries derived OTUs 213 and 214, on the other hand were shared by multiple groups, that were *Thermoplasmatales*, *Methanosaeta*, ANME-2d, *Aenigmarchaeota* and a crenarchaeotic unidentified ("others") group. Further abundant at incubation start was the OTU 175 (176 in 16S rRNA clone library) that was assigned to *Thermoplasmatales*, *Miscellaneous Euryarchaeotic Group* or *Miscellaneous Crenarchaeotic Group*. The OTU pairs 181/182 and 266/267 were assigned to the crenarchaeotic unidentified ('others') group, whereas both 236/237 and 238/238 fell into *Miscellaneous Crenarchaeotic Group.* Overall, a relatively big part of the archaeal community was identified for the incubation start, with 69 % and 77 % for the core and slurry, respectively, whereas several OTUs could not distinctively be assigned.

In the $^{13}CH_4$ incubation, there was a substantial difference in the archaeal communities between the ^{13}C and the ^{12}C fraction regarding occurrences and relative abundances of OTUs. The ^{13}C peak showed a clear dominance of the measured OTUs 213, 212, 175 and 176, which reached a total relative abundance of 77 %. The measured dominance of OTUs 213 and 212 matched the calculated OTUs obtained from sequences from 16S rRNA clone libraries and can be most likely taxonomically identified as members falling into the group ANME-2d. 16S rRNA sequences with OTUs 176 and 177 fell into the groups *Thermoplasmatales*, *Miscellaneous Euryarchaerotic Group* and *Miscellaneous Crenarchaeotic Group*. The OTUs 314, 266, 238, 153, 135 showed relative abundances of <5% each.

The T-RFLP analysis of the ^{12}C peak of the same incubation showed a dominance of the OTUs 213, 212, 93 and 238, which covered together a relative abundance of 72 and 69 % respectively and were taxonomically assigned as described

above. The OTUs 153, 102, 271, 244 and 63 showed contributions to the relative abundance of 2-9 % each.

In the $^{13}C_{DIC}$ slurry incubation, the ^{13}C peak was clearly dominated by the OTUs 213 and 153, which reflected together 65 % of the relative abundances. As described above, the T-RFLP-derived OTU 213 can be assigned to 16S rRNA sequences of the groups *Thermoplasmatales, Methanosaeta, ANME-2d* and *Aenigmarchaeotia*, whereas OTU 153 could not be identified. The OTUs 161, 167, 175, 217, 238 and 314 showed relative abundances of 4-7 % each, but only OTU 238 could be aligned to a sequence with the OTU 239 obtained from the 16S rRNA clone library and identified as a member of *Miscellaneous Crenarchaeotic Group*. As in the ^{13}C peak of the $^{13}CH_4$ sample, also in the ^{13}C peak of the $^{13}C_{DIC}$ sample, OTU 93 was not detected in the T-RFLP analyses. In contrast, in the ^{12}C peak of the $^{13}C_{DIC}$ incubation, OTU 93 could be assigned to *Miscellaneous Crenarchaeotic Group* and was dominating with representing 43 % of the total archaeal community. Other dominating OTUs in this sample were OTU 213 and 212 representing 7 to 26 %, followed by 153, 238, 102, 171 and 233, which contributed to the relative abundance with 4-9 %.

The results of the T-RFLP analysis suggested that more taxonomical units may potentially assimilate methane and/or bicarbonate than indicated by the 16S rRNA clone libraries or that cross-feeding of ^{13}C-labeled metabolites occurred.

3 Identified archaea in AOM zone

The 16S rRNA clone libraries and the T-RFLP analysis provided further information about diversity and identities of archaea that were – besides members of ANME-2d – active in the AOM zone of Lake Ørn sediment. The identified groups were (1) *Thermoplasmatales*, (2) *Methanosaeta*, (3) *Methanolinea* and (4) *Miscellaneous Crenarchaeotic Group*.

The linage *Thermoplasmatales* showed a high contribution to the RNA pool at incubation start in the AOM zone of Lake Ørn sediment. Previously, *Thermoplasmatales* were found in numerous freshwater and marine habitats (e.g., Webster et al., 2011) but their metabolisms are largely unknown and likely as diverse as the group itself. Due to its occurrence in AOM-active areas, a subgroup of *Thermoplasmatales*, named *Marine Benthic Group D* was suggested to be involved in

methane cycling (Beal et al., 2009; Schubert et al., 2011; Webster et al., 2011). Another diverse and widespread genus in methanogenic environments is *Methanosaeta,* a group of obligate aceticlastic methanogens (Mori et al., 2012). *Methanosaeta* has never been shown to hold the capacity to oxidize methane, but members of the group own the complete complement of genes necessary for the reduction of carbon dioxide to methane (Rotaru et al., 2014) and is therefore likely to be part of the methanogenic community in Lake Ørn. Members of the novel family *Methanolinea* were classified as H_2/CO_2-using methanogens and were found in various anaerobic environments, including lake sediment (Bräuer et al., 2006; Sakai et al., 2009, 2012). Even though *Methanolinea* were not retrieved in samples from the incubation start, the groups' activity was represented by two 16S rRNA sequences that were retrieved from the ^{13}C fraction of the $^{13}C_{DIC}$ incubation. Members of *Miscellaneous Crenarchaeotic Group* were found in high amounts at incubation start and a significant amount of ^{13}C-labeled sequences of the group were retrieved from the $^{13}C_{DIC}$ incubations. Genomes of phylum members of this group contain genes necessary for methane metabolism and anaerobic methane oxidation was proposed as possible metabolism due to significant abundances in AOM-active environments (Evans et al., 2015). In Lake Ørn, the ^{13}C-labeling of sequences assigned to *Miscellaneous Crenarchaeotic Group* indicated autotrophy.

4 References

Beal, E. J., House, C. H., and Orphan, V. J. (2009). Manganese- and iron-dependent marine methane oxidation. *Science (80-.).* 325, 184–187. doi:10.1126/science.1169984.

Bräuer, S. L., Cadillo-Quiroz, H., Yashiro, E., Yavitt, J. B., and Zinder, S. H. (2006). Isolation of a novel acidiphilic methanogen from an acidic peat bog. *Nature* 442, 192–194. doi:10.1038/nature04810.

Evans, P. N., Parks, D. H., Chadwick, G. L., Robbins, S. J., Orphan, V. J., Golding, S. D., et al. (2015). Methane metabolism in the archaeal phylum *Bathyarchaeota* revealed by genome-centric metagenomics. *Sci. Reports* 350, 434–438.

Kaplan, C. W., and Kitts, C. L. (2003). Variation between observed and true Terminal Restriction Fragment length is dependent on true TRF length and purine content. *J. Microbiol. Methods* 54, 121–125. doi:10.1016/S0167-7012(03)00003-4.

Moeslund, L., Thamdrup, B., and Jørgensen, B. B. (1994). Sulfur and iron cycling in a coastal sediment: Radiotracer studies and seasonal dynamics. *Biogeochemistry* 27, 129–152.

Mori, K., Lino, T., Suzuki, K.-I., Yamaguchi, K., and Kamagata, Y. (2012). Aceticlastic and NaCl-requiring methanogen *Methanosaeta pelagica* sp. nov., isolated from marine tidal flat sediment. *Appl. Environ. Microbiol.* 78, 3416–3423. doi:10.1128/AEM.07484-11.

Norði, K. Á., Thamdrup, B., and Schubert, C. J. (2013). Anaerobic oxidation of methane in an iron-rich Danish freshwater lake sediment. *Limnol. Oceanogr.* 58, 546–554. doi:10.4319/lo.2013.58.2.0546.

Rotaru, A.-E., Shrestha, P. M., Liu, F., Shrestha, M., Shrestha, D., Embree, M., et al. (2014). A new model for electron flow during anaerobic digestion: direct interspecies electron transfer to *Methanosaeta* for the reduction of carbon dioxide to methane. *Energy Environ. Sci.* 7, 408–415. doi:10.1039/c3ee42189a.

Sakai, S., Ehara, M., Tseng, I.-C., Yamaguchi, T., Bräuer, S. L., Cadillo-Quiroz, H., et al. (2012). *Methanolinea mesophila* sp. nov., a hydrogenotrophic methanogen isolated from rice field soil, and proposal of the archaeal family *Methanoregulaceae* fam. nov. within the order *Methanomicrobiales. Int. J. Syst. Evol. Microbiol.* 62, 1389–1395. doi:10.1099/ijs.0.035048-0.

Sakai, S., Imachi, H., Sekiguchi, Y., Tseng, I.-C., Ohashi, A., Harada, H., et al. (2009). Cultivation of methanogens under low-hydrogen conditions by using the coculture method. *Appl. Environ. Microbiol.* 75, 4892–4896. doi:10.1128/AEM.02835-08.

Schubert, C. J., Vazquez, F., Lösekann-Behrens, T., Knittel, K., Tonolla, M., and Boetius, A. (2011). Evidence for anaerobic oxidation of methane in sediments of a freshwater system (Lago Di Cadagno). *FEMS Microbiol. Ecol.* 76, 26–38. doi:10.1111/j.1574-6941.2010.01036.x.

Webster, G., Sass, H., Cragg, B. A., Gorra, R., Knab, N. J., Green, C. J., et al. (2011). Enrichment and cultivation of prokaryotes associated with the sulphate-methane transition zone of diffusion-controlled sediments of Aarhus Bay, Denmark, under heterotrophic conditions. *FEMS Microbiol. Ecol.* 77, 248–263. doi:10.1111/j.1574-6941.2011.01109.x.

5 Figures and Tables

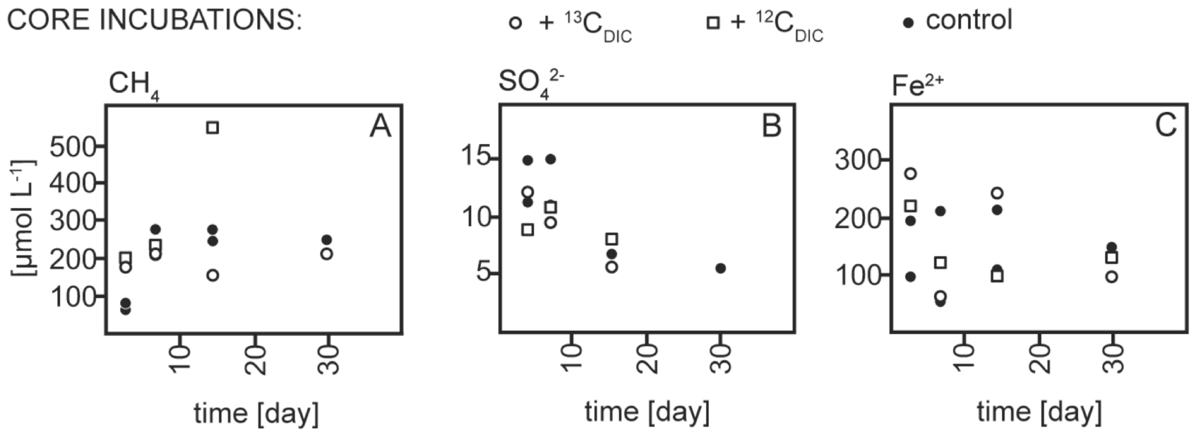

Figure S1. Concentrations over time of **(A)** methane (CH_4), **(B)** sulfate (SO_4^{2-}), and **(C)** dissolved iron (Fe^{2+}) in $^{13}C_{DIC}$, $^{12}C_{DIC}$ and control core incubations.

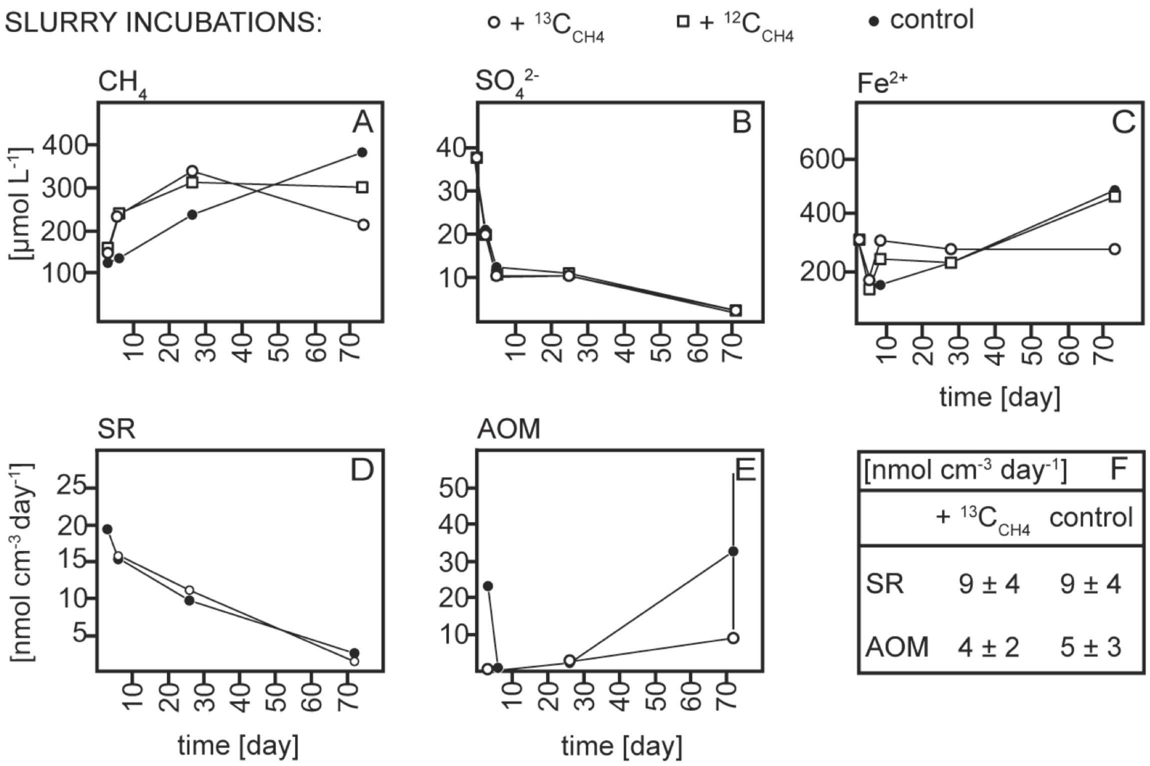

Figure S2. Concentrations over time of **(A)** methane (CH_4), **(B)** sulfate (SO_4^{2-}), and **(C)** dissolved iron (Fe^{2+}), rates over time of **(D)** sulfate reduction (SR), and **(E)** anaerobic methane oxidation (AOM) in $^{13}C_{CH4}$, $^{12}C_{CH4}$ and control slurry incubations and **(F)** average rates of SR and AOM in $^{13}C_{CH4}$ and control slurry incubations.

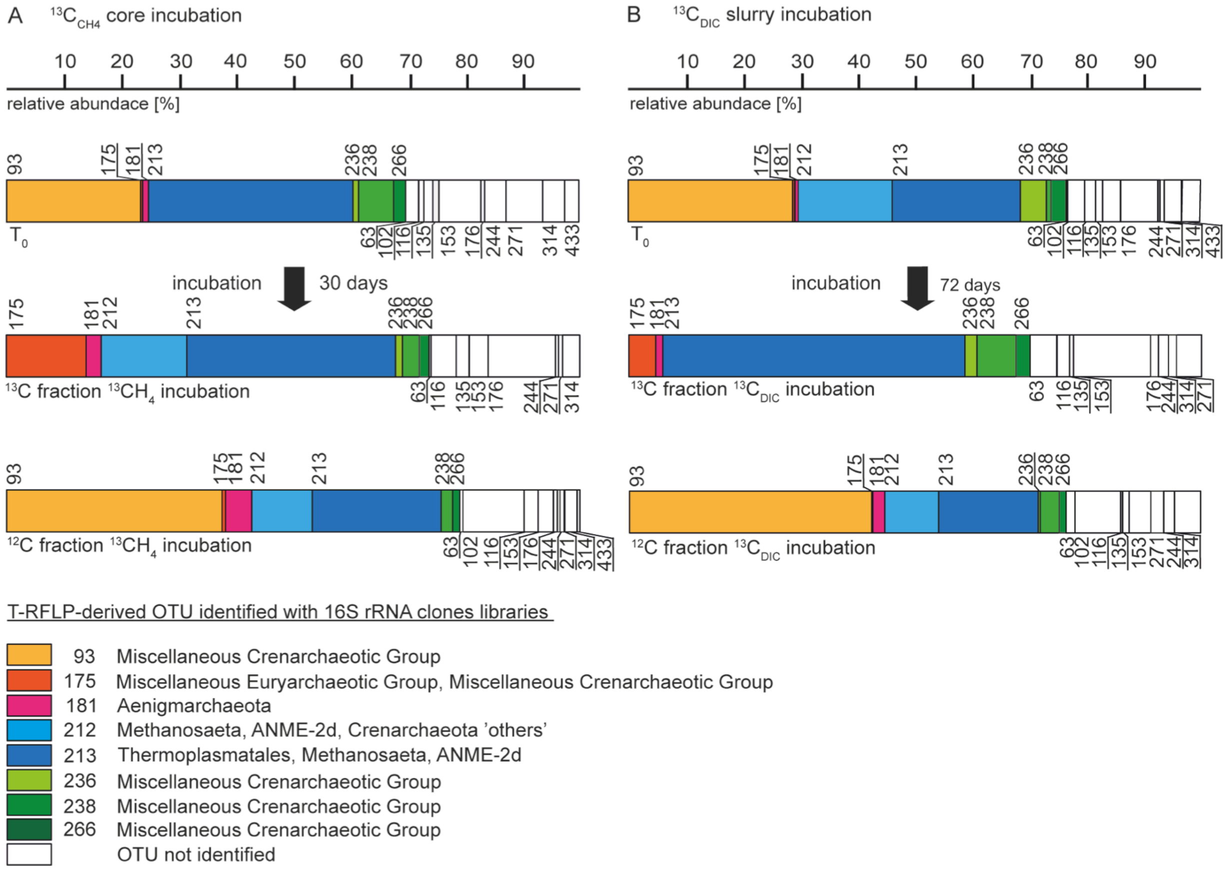

Figure S3. Relative abundances (%) of Operational Taxonomic Units (OTUs) obtained from T-RFLP analysis and OTU identification with 16S rRNA clone libraries: **(A)** T_0, ^{13}C and ^{12}C fraction of $^{13}C_{CH4}$ core incubation and **(B)** T_0, ^{13}C and ^{12}C fraction of $^{13}C_{DIC}$ slurry incubation.

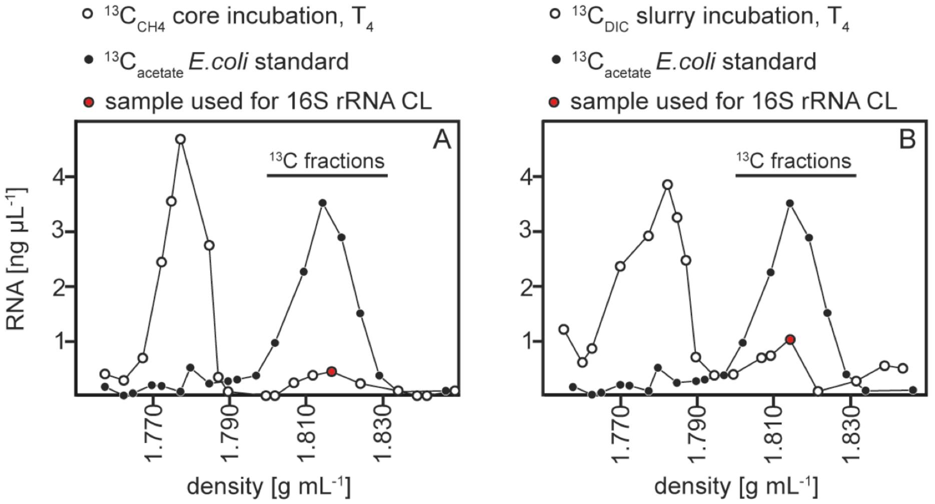

Figure S4: Caesium trifluoroacetate (CsTFA) density gradients of **(A)** $^{13}C_{CH4}$ core incubation at T_4 with $^{13}C_{acetate}$ *E.coli* standard and **(B)** $^{13}C_{DIC}$ slurry incubation at T_4 with $^{13}C_{acetate}$ *E.coli* standard; fractions indicated by red circles were used to construct 16S rRNA clone libraries.

Table S1. Taxonomic affiliation, numbers and operational taxonomical units (OTUs) of sequences obtained from 16S rRNA clone libraries at incubation start (T_0) for one representative sample of $^{13}C_{CH4}$ and $^{13}C_{DIC}$ incubations, each from the ^{13}C fraction and the ^{12}C fraction of the same CsTFA gradients.

**Taxonomic affiliation**	**OTU**	**T_0**	**$^{13}CH_4$ core**		**$^{13}C_{DIC}$ slurry**	
			**^{13}C**	**^{12}C**	**^{13}C**	**^{12}C**
EURYARCHAEOTA	21	3		1		
Thermoplasmata	69			1	1	
Thermoplasmatales	70	1		1		
	155	5				1
	156	1		1		1
	176	1				
	180					1
	206					1
	207	1				
	209	1				
	214	4		3	1	1
	n.i.	2		1		1
Methanomicrobia	213	2	2	1	3	
Methanosarcinales	214		1			
Methanosaetacea	n.i.	1				
Methanosaeta						
Methanomicrobia	213		3			
Methanosarcinales	214	6	16	1	16	
ANME-2d	215		2			
	n.i.	3	1		1	
Methanomicrobia	21				2	
Methanomicrobiales						
Methanoregulacea/Methanolinea						
MISCELLANEOUS EURYARCHAEOTIC GROUP	159			1		
	176			1		
	288	1		3		3
	328					1
	330			1		
	n.i.	1				1
AENIGMARCHAEOTA	181					1
	195	1				1
	154			1		
	215			1		
	485					1
	n.i.	1				
CRENARCHAEOTA						
Marine Benthic Group B	292		1			
others	182	1				
	213	1				
	243			1		
	267					1
MISCELLANEOUS CRENARCHAEOTIC GROUP	94					1
	151	1				
	174	1		1		2
	176			1		
	237	4				1
	238	3		3		3
	239	2	1	8	3	7
	240	1		1		1
	241	1		1		
	242			1		
	244	4		1		1
	266	1				
	268	1				
	270					2
	271	1		2		1
	297				1	
	342					1
	n.i.	20		2	1	1

Table S2. Relative abundances (%) of Operational Taxonomical Units (OTUs) obtained from T-RFLP analysis at incubation start (T_0 core and slurry) and for one representative sample of the ^{13}C fraction and the ^{12}C fraction of the same CsTFA gradient of the $^{13}C_{CH4}$ and $^{13}C_{DIC}$ incubations, respectively.

**OTU**	T_0 core (%)	T_0 slurry (%)	$^{13}C_{CH4}$ fraction $^{13}C_{CH4}$ incubation (%)	$^{12}C_{CH4}$ fraction $^{13}C_{CH4}$ incubation (%)	$^{13}C_{DIC}$ fraction $^{13}C_{DIC}$ incubation (%)	$^{12}C_{DIC}$ fraction $^{13}C_{DIC}$ incubation (%)
**116**	2	1	4	0	4	0
**135**	1	3	2	0	1	1
**181**	1	0	3	5	1	2
**433**	3	3	0	2	0	0
**244**	4	0	0	1	2	2
**236**	1	5	1	0	2	0
**63**	2	3	0	1	0	2
**266**	2	3	2	1	2	1
**176**	0	0	12	1	5	0
**175**	0	0	14	1	5	0
**271**	7	1	1	1	3	4
**314**	4	3	3	2	4	3
**102**	1	2	0	11	0	8
**238**	6	1	3	2	7	4
**153**	7	7	3	3	13	4
**212**	0	16	15	11	0	10
**93**	23	29	0	37	0	43
**213**	35	23	36	22	51	16

Table S3: Gene Bank Identification of Lake Ørn 16S rRNA sequences: sequence IDs, clone IDs, organism, incubation/treatment/source and accession number.

Sequence ID	Clone ID	Organism	Incubation/treatment/source	Accession number
T0core_1	C1	uncultured Crenarchaeote	T0 core	KX463203
T0core_2	C2	uncultured Crenarchaeote	T0 core	KX463211
T0core_3	C3	uncultured Thermoplasmatales	T0 core	KX463217
T0core_5	C5	uncultured Crenarchaeote	T0 core	KX463227
T0core_6	C6	uncultured Crenarchaeote	T0 core	KX463228
T0core_7	C7	uncultured Crenarchaeote	T0 core	KX463229
T0core_8	C8	uncultured Crenarchaeote	T0 core	KX463230
T0core_10	C10	uncultured Crenarchaeote	T0 core	KX463195
T0core_12	C12	uncultured Crenarchaeote	T0 core	KX463196
T0core_13	C13	uncultured Crenarchaeote	T0 core	KX463197
T0core_14	C14	uncultured Thermoplasmatales	T0 core	KX463198
T0core_15	C15	uncultured Crenarchaeote	T0 core	KX463199
T0core_16	C16	uncultured Crenarchaeote	T0 core	KX463200
T0core_18	C18	uncultured Crenarchaeote	T0 core	KX463201
T0core_19	C19	uncultured Crenarchaeote	T0 core	KX463202
T0core_22	C22	uncultured Crenarchaeote	T0 core	KX463204
T0core_23	C23	uncultured Thermoplasmatales	T0 core	KX463205
T0core_24	C24	uncultured Crenarchaeote	T0 core	KX463206
T0core_25	C25	uncultured Crenarchaeote	T0 core	KX463207
T0core_27	C27	uncultured Thermoplasmatales	T0 core	KX463208
T0core_28	C28	uncultured Thermoplasmatales	T0 core	KX463209
T0core_29	C29	uncultured Crenarchaeote	T0 core	KX463210
T0core_31	C31	uncultured Archaeon	T0 core	KX463212
T0core_32	C32	uncultured Thermoplasmatales	T0 core	KX463213
T0core_33	C33	uncultured Thermoplasmatales	T0 core	KX463214
T0core_38	C38	uncultured Thermoplasmatales	T0 core	KX463215

T0core_39	C39	uncultured Thermoplasmatales	T0 core	KX463216
T0core_43	C43	uncultured Crenarchaeote	T0 core	KX463218
T0core_44	C44	ANME-2d cluster	T0 core	KX463219
T0core_45	C45	uncultured Crenarchaeote	T0 core	KX463220
T0core_46	C46	uncultured Crenarchaeote	T0 core	KX463221
T0core_47	C47	uncultured Crenarchaeote	T0 core	KX463222
T0core_48	C48	uncultured Crenarchaeote	T0 core	KX463223
T0core_51	C51	uncultured Archaeon	T0 core	KX463224
T0core_52	C52	uncultured Thermoplasmatales	T0 core	KX463225
T0core_54	C54	uncultured Thermoplasmatales	T0 core	KX463226
T0slurry_1	S1	uncultured Thermoplasmatales	T0 slurry	KX463239
T0slurry_3	S3	ANME-2d cluster	T0 slurry	KX463253
T0slurry_4	S4	uncultured Methanosaeta	T0 slurry	KX463262
T0slurry_5	S5	ANME-2d cluster	T0 slurry	KX463267
T0slurry_6	S6	uncultured Crenarchaeote	T0 slurry	KX463269
T0slurry_10	S10	uncultured Crenarchaeote	T0 slurry	KX463231
T0slurry_12	S12	uncultured Crenarchaeote	T0 slurry	KX463232
T0slurry_13	S13	ANME-2d cluster	T0 slurry	KX463233
T0slurry_14	S14	uncultured Thermoplasmatales	T0 slurry	KX463234
T0slurry_15	S15	uncultured Crenarchaeote	T0 slurry	KX463235
T0slurry_16	S16	uncultured Archaeon	T0 slurry	KX463236
T0slurry_17	S17	uncultured Crenarchaeote	T0 slurry	KX463237
T0slurry_19	S19	uncultured Crenarchaeote	T0 slurry	KX463238
T0slurry_20	S20	uncultured Archaeon	T0 slurry	KX463240
T0slurry_21	S21	ANME-2d cluster	T0 slurry	KX463241
T0slurry_23	S23	uncultured Thermoplasmatales	T0 slurry	KX463242
T0slurry_24	S24	uncultured Thermoplasmatales	T0 slurry	KX463243
T0slurry_28	S28	uncultured Crenarchaeote	T0 slurry	KX463244

T0slurry_29	S29	uncultured Methanosaeta	T0 slurry	KX463245
T0slurry_30	S30	uncultured Crenarchaeote	T0 slurry	KX463246
T0slurry_31	S31	uncultured Thermoplasmatales	T0 slurry	KX463247
T0slurry_33	S33	uncultured Crenarchaeote	T0 slurry	KX463248
T0slurry_35	S35	ANME-2d cluster	T0 slurry	KX463249
T0slurry_36	S36	ANME-2d cluster	T0 slurry	KX463250
T0slurry_38	S38	uncultured Archaeon	T0 slurry	KX463251
T0slurry_39	S39	uncultured Crenarchaeote	T0 slurry	KX463252
T0slurry_42	S42	uncultured Methanosaeta	T0 slurry	KX463254
T0slurry_43	S43	uncultured Crenarchaeote	T0 slurry	KX463255
T0slurry_44	S44	uncultured Crenarchaeote	T0 slurry	KX463256
T0slurry_45	S45	ANME-2d cluster	T0 slurry	KX463257
T0slurry_46	S46	uncultured Crenarchaeote	T0 slurry	KX463258
T0slurry_47	S47	uncultured Archaeon	T0 slurry	KX463259
T0slurry_48	S48	uncultured Thermoplasmatales	T0 slurry	KX463260
T0slurry_49	S49	uncultured Thermoplasmatales	T0 slurry	KX463261
T0slurry_51	S51	uncultured Crenarchaeote	T0 slurry	KX463263
T0slurry_53	S53	uncultured Crenarchaeote	T0 slurry	KX463264
T0slurry_55	S55	uncultured Archaeon	T0 slurry	KX463265
T0slurry_59	S59	uncultured Crenarchaeote	T0 slurry	KX463266
T0slurry_60	S60	uncultured Crenarchaeote	T0 slurry	KX463268
13Cpeak_13CH4incub_3	AA3	ANME-2d cluster	^{13}C peak in $^{13}CH_4$ incubation	KX463072
13Cpeak_13CH4incub_5	AA5	ANME-2d cluster	^{13}C peak in $^{13}CH_4$ incubation	KX463077
13Cpeak_13CH4incub_10	AA10	uncultured Archaeon	^{13}C peak in $^{13}CH_4$ incubation	KX463063
13Cpeak_13CH4incub_12	AA12	ANME-2d cluster	^{13}C peak in $^{13}CH_4$ incubation	KX463064
13Cpeak_13CH4incub_18	AA18	ANME-2d cluster	^{13}C peak in $^{13}CH_4$ incubation	KX463065
13Cpeak_13CH4incub_26	AA26	ANME-2d cluster	^{13}C peak in $^{13}CH_4$ incubation	KX463066
13Cpeak_13CH4incub_30	AA30	ANME-2d cluster	^{13}C peak in $^{13}CH_4$ incubation	KX463067

13Cpeak_13CH4incub_31	AA31	uncultured Methanosaeta	^{13}C peak in $^{13}CH_4$ incubation	KX463068
13Cpeak_13CH4incub_32	AA32	ANME-2d cluster	^{13}C peak in $^{13}CH_4$ incubation	KX463069
13Cpeak_13CH4incub_35	AA35	ANME-2d cluster	^{13}C peak in $^{13}CH_4$ incubation	KX463070
13Cpeak_13CH4incub_37	AA37	uncultured Crenarchaeote	^{13}C peak in $^{13}CH_4$ incubation	KX463071
13Cpeak_13CH4incub_41	AA41	ANME-2d cluster	^{13}C peak in $^{13}CH_4$ incubation	KX463073
13Cpeak_13CH4incub_45	AA45	ANME-2d cluster	^{13}C peak in $^{13}CH_4$ incubation	KX463074
13Cpeak_13CH4incub_46	AA46	ANME-2d cluster	^{13}C peak in $^{13}CH_4$ incubation	KX463075
13Cpeak_13CH4incub_53	AA53	ANME-2d cluster	^{13}C peak in $^{13}CH_4$ incubation	KX463076
13Cpeak_13CH4incub_63	AA63	ANME-2d cluster	^{13}C peak in $^{13}CH_4$ incubation	KX463078
13Cpeak_13CH4incub_64	AA64	ANME-2d cluster	^{13}C peak in $^{13}CH_4$ incubation	KX463079
13Cpeak_13CH4incub_65	AA56	ANME-2d cluster	^{13}C peak in $^{13}CH_4$ incubation	KX463080
13Cpeak_13CH4incub_71	AA71	ANME-2d cluster	^{13}C peak in $^{13}CH_4$ incubation	KX463081
13Cpeak_13CH4incub_72	AA72	ANME-2d cluster	^{13}C peak in $^{13}CH_4$ incubation	KX463082
13Cpeak_13CH4incub_74	AA74	uncultured Methanosaeta	^{13}C peak in $^{13}CH_4$ incubation	KX463083
13Cpeak_13CH4incub_80	AA80	ANME-2d cluster	^{13}C peak in $^{13}CH_4$ incubation	KX463084
13Cpeak_13CH4incub_84	AA84	ANME-2d cluster	^{13}C peak in $^{13}CH_4$ incubation	KX463085
13Cpeak_13CH4incub_89	AA89	ANME-2d cluster	^{13}C peak in $^{13}CH_4$ incubation	KX463086
13Cpeak_13CH4incub_91	AA91	ANME-2d cluster	^{13}C peak in $^{13}CH_4$ incubation	KX463087
13Cpeak_13CH4incub_93	AA93	ANME-2d cluster	^{13}C peak in $^{13}CH_4$ incubation	KX463088
13Cpeak_13CH4incub_94	AA94	uncultured Methanosaeta	^{13}C peak in $^{13}CH_4$ incubation	KX463089
12Cpeak_13CH4incub_2	AB2	uncultured Thermoplasmatales	^{12}C peak in $^{13}CH_4$ incubation	KX463105
12Cpeak_13CH4incub_3	AB3	uncultured Crenarchaeote	^{12}C peak in $^{13}CH_4$ incubation	KX463116
12Cpeak_13CH4incub_4	AB4	uncultured Crenarchaeote	^{12}C peak in $^{13}CH_4$ incubation	KX463122
12Cpeak_13CH4incub_5	AB5	Uncultured Archaeon	^{12}C peak in $^{13}CH_4$ incubation	KX463126
12Cpeak_13CH4incub_7	AB7	uncultured Crenarchaeote	^{12}C peak in $^{13}CH_4$ incubation	KX463127
12Cpeak_13CH4incub_8	AB8	uncultured Methanosaeta	^{12}C peak in $^{13}CH_4$ incubation	KX463128
12Cpeak_13CH4incub_9	AB9	uncultured Crenarchaeote	^{12}C peak in $^{13}CH_4$ incubation	KX463129
12Cpeak_13CH4incub_10	AB10	uncultured Archaeon	^{12}C peak in $^{13}CH_4$ incubation	KX463090

12Cpeak_13CH4incub_11	AB11	uncultured Archaeon	^{12}C peak in $^{13}CH_4$ incubation	KX463091
12Cpeak_13CH4incub_12	AB12	uncultured Crenarchaeote	^{12}C peak in $^{13}CH_4$ incubation	KX463092
12Cpeak_13CH4incub_13	AB13	uncultured Archaeon	^{12}C peak in $^{13}CH_4$ incubation	KX463093
12Cpeak_13CH4incub_16	AB16	uncultured Archaeon	^{12}C peak in $^{13}CH_4$ incubation	KX463094
12Cpeak_13CH4incub_17	AB17	uncultured Crenarchaeote	^{12}C peak in $^{13}CH_4$ incubation	KX463095
12Cpeak_13CH4incub_18	AB18	uncultured Archaeon	^{12}C peak in $^{13}CH_4$ incubation	KX463096
12Cpeak_13CH4incub_19	AB19	uncultured Crenarchaeote	^{12}C peak in $^{13}CH_4$ incubation	KX463097
12Cpeak_13CH4incub_20	AB20	uncultured Archaeon	^{12}C peak in $^{13}CH_4$ incubation	KX463098
12Cpeak_13CH4incub_21	AB21	uncultured Crenarchaeote	^{12}C peak in $^{13}CH_4$ incubation	KX463099
12Cpeak_13CH4incub_23	AB23	uncultured Crenarchaeote	^{12}C peak in $^{13}CH_4$ incubation	KX463100
12Cpeak_13CH4incub_25	AB25	uncultured Thermoplasmatales	^{12}C peak in $^{13}CH_4$ incubation	KX463101
12Cpeak_13CH4incub_26	AB26	uncultured Crenarchaeote	^{12}C peak in $^{13}CH_4$ incubation	KX463102
12Cpeak_13CH4incub_27	AB27	uncultured Crenarchaeote	^{12}C peak in $^{13}CH_4$ incubation	KX463103
12Cpeak_13CH4incub_28	AB28	uncultured Thermoplasmatales	^{12}C peak in $^{13}CH_4$ incubation	KX463104
12Cpeak_13CH4incub_30	AB30	uncultured Crenarchaeote	^{12}C peak in $^{13}CH_4$ incubation	KX463106
12Cpeak_13CH4incub_31	AB31	uncultured Thermoplasmatales	^{12}C peak in $^{13}CH_4$ incubation	KX463107
12Cpeak_13CH4incub_32	AB32	uncultured Crenarchaeote	^{12}C peak in $^{13}CH_4$ incubation	KX463108
12Cpeak_13CH4incub_33	AB33	uncultured Crenarchaeote	^{12}C peak in $^{13}CH_4$ incubation	KX463109
12Cpeak_13CH4incub_34	AB34	uncultured Crenarchaeote	^{12}C peak in $^{13}CH_4$ incubation	KX463110
12Cpeak_13CH4incub_35	AB35	uncultured Archaeon	^{12}C peak in $^{13}CH_4$ incubation	KX463111
12Cpeak_13CH4incub_36	AB36	uncultured Crenarchaeote	^{12}C peak in $^{13}CH_4$ incubation	KX463112
12Cpeak_13CH4incub_37	AB37	uncultured Thermoplasmatales	^{12}C peak in $^{13}CH_4$ incubation	KX463113
12Cpeak_13CH4incub_38	AB38	uncultured Thermoplasmatales	^{12}C peak in $^{13}CH_4$ incubation	KX463114
12Cpeak_13CH4incub_39	AB39	uncultured Thermoplasmatales	^{12}C peak in $^{13}CH_4$ incubation	KX463115
12Cpeak_13CH4incub_40	AB40	uncultured Archaeon	^{12}C peak in $^{13}CH_4$ incubation	KX463117
12Cpeak_13CH4incub_41	AB41	uncultured Crenarchaeote	^{12}C peak in $^{13}CH_4$ incubation	KX463118
12Cpeak_13CH4incub_42	AB42	uncultured Crenarchaeote	^{12}C peak in $^{13}CH_4$ incubation	KX463119
12Cpeak_13CH4incub_44	AB44	uncultured Archaeon	^{12}C peak in $^{13}CH_4$ incubation	KX463120

12Cpeak_13CH4incub_47	AB47	uncultured Thermoplasmatales	^{12}C peak in $^{13}CH_4$ incubation	KX463121
12Cpeak_13CH4incub_50	AB50	uncultured Crenarchaeote	^{12}C peak in $^{13}CH_4$ incubation	KX463123
12Cpeak_13CH4incub_52	AB52	uncultured Crenarchaeote	^{12}C peak in $^{13}CH_4$ incubation	KX463124
12Cpeak_13CH4incub_53	AB53	ANME-2d cluster	^{12}C peak in $^{13}CH_4$ incubation	KX463125
13Cpeak_13CDICincub_7	BA7	ANME-2d cluster	^{13}C peak in $^{13}C_{DIC}$ incubation	KX463153
13Cpeak_13CDICincub _10	BA10	ANME-2d cluster	^{13}C peak in $^{13}C_{DIC}$ incubation	KX463130
13Cpeak_13CDICincub _11	BA11	uncultured Methanosaeta	^{13}C peak in $^{13}C_{DIC}$ incubation	KX463131
13Cpeak_13CDICincub _16	BA16	uncultured Methanosaeta	^{13}C peak in $^{13}C_{DIC}$ incubation	KX463132
13Cpeak_13CDICincub _25	BA25	ANME-2d cluster	^{13}C peak in $^{13}C_{DIC}$ incubation	KX463133
13Cpeak_13CDICincub _28	BA28	ANME-2d cluster	^{13}C peak in $^{13}C_{DIC}$ incubation	KX463134
13Cpeak_13CDICincub _30	BA30	uncultured Crenarchaeote	^{13}C peak in $^{13}C_{DIC}$ incubation	KX463135
13Cpeak_13CDICincub _31	BA31	ANME-2d cluster	^{13}C peak in $^{13}C_{DIC}$ incubation	KX463136
13Cpeak_13CDICincub _32	BA32	ANME-2d cluster	^{13}C peak in $^{13}C_{DIC}$ incubation	KX463137
13Cpeak_13CDICincub _34	BA34	ANME-2d cluster	^{13}C peak in $^{13}C_{DIC}$ incubation	KX463138
13Cpeak_13CDICincub _39	BA39	uncultured Crenarchaeote	^{13}C peak in $^{13}C_{DIC}$ incubation	KX463139
13Cpeak_13CDICincub _40	BA40	ANME-2d cluster	^{13}C peak in $^{13}C_{DIC}$ incubation	KX463140
13Cpeak_13CDICincub _43	BA43	ANME-2d cluster	^{13}C peak in $^{13}C_{DIC}$ incubation	KX463141
13Cpeak_13CDICincub _49	BA49	uncultured Crenarchaeote	^{13}C peak in $^{13}C_{DIC}$ incubation	KX463142
13Cpeak_13CDICincub _50	BA50	uncultured Methanolinea	^{13}C peak in $^{13}C_{DIC}$ incubation	KX463143
13Cpeak_13CDICincub _55	BA55	ANME-2d cluster	^{13}C peak in $^{13}C_{DIC}$ incubation	KX463144
13Cpeak_13CDICincub _56	BA56	uncultured Thermoplasmatales	^{13}C peak in $^{13}C_{DIC}$ incubation	KX463145
13Cpeak_13CDICincub _65	BA65	uncultured Methanolinea	^{13}C peak in $^{13}C_{DIC}$ incubation	KX463146
13Cpeak_13CDICincub _67	BA67	ANME-2d cluster	^{13}C peak in $^{13}C_{DIC}$ incubation	KX463147
13Cpeak_13CDICincub _69	BA69	ANME-2d cluster	^{13}C peak in $^{13}C_{DIC}$ incubation	KX463148
13Cpeak_13CDICincub _74	BA74	ANME-2d cluster	^{13}C peak in $^{13}C_{DIC}$ incubation	KX463149
13Cpeak_13CDICincub _75	BA75	uncultured Crenarchaeote	^{13}C peak in $^{13}C_{DIC}$ incubation	KX463150
13Cpeak_13CDICincub _76	BA76	ANME-2d cluster	^{13}C peak in $^{13}C_{DIC}$ incubation	KX463151
13Cpeak_13CDICincub _79	BA79	uncultured Methanosaeta	^{13}C peak in $^{13}C_{DIC}$ incubation	KX463152

13Cpeak_13CDICincub _80	BA80	ANME-2d cluster	^{13}C peak in $^{13}C_{DIC}$ incubation	KX463154
13Cpeak_13CDICincub _84	BA84	ANME-2d cluster	^{13}C peak in $^{13}C_{DIC}$ incubation	KX463155
13Cpeak_13CDICincub _89	BA89	ANME-2d cluster	^{13}C peak in $^{13}C_{DIC}$ incubation	KX463156
13Cpeak_13CDICincub _90	BA90	uncultured Crenarchaeote	^{13}C peak in $^{13}C_{DIC}$ incubation	KX463157
13Cpeak_13CDICincub _95	BA95	uncultured Thermoplasmatales	^{13}C peak in $^{13}C_{DIC}$ incubation	KX463158
12Cpeak_13CDICincub _5	BB5	uncultured Crenarchaeote	^{12}C peak in $^{13}C_{DIC}$ incubation	KX463191
12Cpeak_13CDICincub _7	BB7	uncultured Crenarchaeote	^{12}C peak in $^{13}C_{DIC}$ incubation	KX463192
12Cpeak_13CDICincub _8	BB8	uncultured Crenarchaeote	^{12}C peak in $^{13}C_{DIC}$ incubation	KX463193
12Cpeak_13CDICincub _9	BB9	uncultured Crenarchaeote	^{12}C peak in $^{13}C_{DIC}$ incubation	KX463194
12Cpeak_13CDICincub _10	BB10	uncultured Thermoplasmatales	^{12}C peak in $^{13}C_{DIC}$ incubation	KX463159
12Cpeak_13CDICincub _12	BB12	uncultured Thermoplasmatales	^{12}C peak in $^{13}C_{DIC}$ incubation	KX463160
12Cpeak_13CDICincub _13	BB13	uncultured Archaeon	^{12}C peak in $^{13}C_{DIC}$ incubation	KX463161
12Cpeak_13CDICincub _14	BB14	uncultured Crenarchaeote	^{12}C peak in $^{13}C_{DIC}$ incubation	KX463162
12Cpeak_13CDICincub _16	BB16	uncultured Crenarchaeote	^{12}C peak in $^{13}C_{DIC}$ incubation	KX463163
12Cpeak_13CDICincub _18	BB18	uncultured Crenarchaeote	^{12}C peak in $^{13}C_{DIC}$ incubation	KX463164
12Cpeak_13CDICincub _20	BB20	uncultured Crenarchaeote	^{12}C peak in $^{13}C_{DIC}$ incubation	KX463165
12Cpeak_13CDICincub _21	BB21	uncultured Crenarchaeote	^{12}C peak in $^{13}C_{DIC}$ incubation	KX463166
12Cpeak_13CDICincub _22	BB22	uncultured Crenarchaeote	^{12}C peak in $^{13}C_{DIC}$ incubation	KX463167
12Cpeak_13CDICincub _23	BB23	uncultured Crenarchaeote	^{12}C peak in $^{13}C_{DIC}$ incubation	KX463168
12Cpeak_13CDICincub _25	BB25	uncultured Crenarchaeote	^{12}C peak in $^{13}C_{DIC}$ incubation	KX463169
12Cpeak_13CDICincub _27	BB27	uncultured Archaeon	^{12}C peak in $^{13}C_{DIC}$ incubation	KX463170
12Cpeak_13CDICincub _29	BB29	uncultured Crenarchaeote	^{12}C peak in $^{13}C_{DIC}$ incubation	KX463171
12Cpeak_13CDICincub _30	BB30	uncultured Archaeon	^{12}C peak in $^{13}C_{DIC}$ incubation	KX463172
12Cpeak_13CDICincub _34	BB34	uncultured Archaeon	^{12}C peak in $^{13}C_{DIC}$ incubation	KX463173
12Cpeak_13CDICincub _37	BB37	uncultured Crenarchaeote	^{12}C peak in $^{13}C_{DIC}$ incubation	KX463174
12Cpeak_13CDICincub _38	BB38	uncultured Crenarchaeote	^{12}C peak in $^{13}C_{DIC}$ incubation	KX463175
12Cpeak_13CDICincub _41	BB41	uncultured Crenarchaeote	^{12}C peak in $^{13}C_{DIC}$ incubation	KX463176
12Cpeak_13CDICincub _42	BB42	uncultured Archaeon	^{12}C peak in $^{13}C_{DIC}$ incubation	KX463177

12Cpeak_13CDICincub _43	BB43	uncultured Archaeon	^{12}C peak in $^{13}C_{DIC}$ incubation	KX463178
12Cpeak_13CDICincub _45	BB45	uncultured Thermoplasmatales	^{12}C peak in $^{13}C_{DIC}$ incubation	KX463179
12Cpeak_13CDICincub _46	BB46	uncultured Archaeon	^{12}C peak in $^{13}C_{DIC}$ incubation	KX463180
12Cpeak_13CDICincub _47	BB47	uncultured Thermoplasmatales	^{12}C peak in $^{13}C_{DIC}$ incubation	KX463181
12Cpeak_13CDICincub _48	BB48	uncultured Archaeon	^{12}C peak in $^{13}C_{DIC}$ incubation	KX463182
12Cpeak_13CDICincub _49	BB49	uncultured Thermoplasmatales	^{12}C peak in $^{13}C_{DIC}$ incubation	KX463183
12Cpeak_13CDICincub _50	BB50	uncultured Crenarchaeote	^{12}C peak in $^{13}C_{DIC}$ incubation	KX463184
12Cpeak_13CDICincub _51	BB51	uncultured Crenarchaeote	^{12}C peak in $^{13}C_{DIC}$ incubation	KX463185
12Cpeak_13CDICincub _54	BB54	uncultured Crenarchaeote	^{12}C peak in $^{13}C_{DIC}$ incubation	KX463186
12Cpeak_13CDICincub _55	BB55	uncultured Thermoplasmatales	^{12}C peak in $^{13}C_{DIC}$ incubation	KX463187
12Cpeak_13CDICincub _56	BB56	uncultured Crenarchaeote	^{12}C peak in $^{13}C_{DIC}$ incubation	KX463188
12Cpeak_13CDICincub _57	BB57	uncultured Archaeon	^{12}C peak in $^{13}C_{DIC}$ incubation	KX463189
12Cpeak_13CDICincub _58	BB58	uncultured Crenarchaeote	^{12}C peak in $^{13}C_{DIC}$ incubation	KX463190
